# Metagenomic and -transcriptomic analyses of microbial nitrogen transformation potential, and gene expression in Swiss lake sediments

**DOI:** 10.1093/ismeco/ycae110

**Published:** 2024-10-15

**Authors:** Kathrin B L Baumann, Alessandra Mazzoli, Guillem Salazar, Hans-Joachim Ruscheweyh, Beat Müller, Robert Niederdorfer, Shinichi Sunagawa, Mark A Lever, Moritz F Lehmann, Helmut Bürgmann

**Affiliations:** Eawag, Swiss Federal Institute of Aquatic Science and Technology, 6047 Kastanienbaum, Switzerland; Department of Environmental Sciences, University of Basel, 4056 Basel, Switzerland; Department of Biology, Institute of Microbiology and Swiss Institute of Bioinformatics, ETH Zurich, 8093 Zurich, Switzerland; Department of Biology, Institute of Microbiology and Swiss Institute of Bioinformatics, ETH Zurich, 8093 Zurich, Switzerland; Eawag, Swiss Federal Institute of Aquatic Science and Technology, 6047 Kastanienbaum, Switzerland; Eawag, Swiss Federal Institute of Aquatic Science and Technology, 6047 Kastanienbaum, Switzerland; Department of Biology, Institute of Microbiology and Swiss Institute of Bioinformatics, ETH Zurich, 8093 Zurich, Switzerland; Institute of Biogeochemistry and Pollutant Dynamics, ETH Zurich, 8092 Zurich, Switzerland; Now at Marine Science Institute, University of Texas at Austin, Port Aransas, 78373 TX, United States; Department of Environmental Sciences, University of Basel, 4056 Basel, Switzerland; Eawag, Swiss Federal Institute of Aquatic Science and Technology, 6047 Kastanienbaum, Switzerland

**Keywords:** denitrification, nitrification, anammox, DNRA, freshwater sediment, metatranscriptomics, metagenomics

## Abstract

The global nitrogen (N) cycle has been strongly altered by anthropogenic activities, including increased input of bioavailable N into aquatic ecosystems. Freshwater sediments are hotspots with regards to the turnover and elimination of fixed N, yet the environmental controls on the microbial pathways involved in benthic N removal are not fully understood. Here, we analyze the abundance and expression of microbial genes involved in N transformations using metagenomics and -transcriptomics across sediments of 12 Swiss lakes that differ in sedimentation rates and trophic regimes. Our results indicate that microbial N loss in these sediments is primarily driven by nitrification coupled to denitrification. N-transformation gene compositions indicated three groups of lakes: agriculture-influenced lakes characterized by rapid depletion of oxidants in the sediment porewater, pristine-alpine lakes with relatively deep sedimentary penetration of oxygen and nitrate, and large, deep lakes with intermediate porewater hydrochemical properties. Sedimentary organic matter (OM) characteristics showed the strongest correlations with the community structure of microbial N-cycling communities. Most transformation pathways were expressed, but expression deviated from gene abundance and did not correlate with benthic geochemistry. Cryptic N-cycling may maintain transcriptional activity even when substrate levels are below detection. Sediments of large, deep lakes generally showed lower in-situ N gene expression than agriculture-influenced lakes, and half of the pristine-alpine lakes. This implies that prolonged OM mineralization in the water column can lead to the suppression of benthic N gene expression.

## Introduction

The nitrogen (N) cycle, driven mainly by microorganisms, is one of Earth’s most dynamic and complex biogeochemical nutrient cycles [1–3]. Natural inputs of bioavailable (i.e., fixed) N in freshwater systems derive primarily from atmospheric deposition, run-off, and biological fixation of dinitrogen (N_2_) [4]. Anthropogenic activities, such as application of fertilizers, combustion of fossil fuels, and discharge of wastewater have doubled the N input to terrestrial and aquatic systems since pre-industrial times [5, 6]. This has led to eutrophication, loss of biodiversity and water quality, anoxia, and alterations in the N cycle [4, 7]. Freshwater sediments are important fixed-N sinks that reduce N export to downstream ecosystems and limit algal blooms in N-limited coastal waters [5]. In lakes, fixed-N turnover rates were shown to increase with increasing N loads, and to correlate positively with mean water residence time, and negatively with mean lake depth [7, 8].

The N cycle is tightly coupled with the carbon (C) cycle, as biomass production requires both C and N, and many of the microbes involved in N transformation are heterotrophs [4]. Similarly, tight links exist with the phosphorous (P) cycle, given that most temperate lakes are P-limited ecosystems [4]. Enhanced primary production due to P input leads to more nitrate (NO_3_^−^) uptake in surface waters, and at the same time to enhanced organic matter (OM) export to sediments, which then fuels denitrification (DN). Indeed, Finlay et al. (2013) reported seven-fold higher N-removal rates in eutrophic compared to oligotrophic lakes [5]. Yet, long-term data from 21 Swiss lakes revealed a more complex picture, indicating that the areal rate of DN is foremost controlled by NO_3_^−^ concentrations in bottom water rather than lake trophic state [8]. Other parameters that likely affect N-removal rates are OM reactivity and quantity, external N input, water residence time, and O_2_ concentrations [4, 5, 8–10].

The microbial N cycle is generally well understood, although new details have emerged in recent years [3, 11]. Under O_2_-depleted conditions, microbes use NO_2_^−^ and NO_3_^−^ as electron acceptors to oxidize OM or other reduced substances (sulfide), ultimately producing N_2_ by complete DN, or NH_4_^+^ by dissimilatory nitrate reduction to ammonium (DNRA) [3, 12–14]. The NO_3_^−^ used for DN or DNRA diffuses into sediments from the overlying water, or is produced in oxic surface sediments by nitrification (i.e., coupled nitrification-DN) [11, 15]. While almost all denitrifiers have the capacity to also respire O_2_ [16–18], DNRA is performed by specific groups of (anaerobic) fermentative and sulfur-oxidizing microorganisms [19]. The rates of both processes depend on a variety of factors, including the availability of NO_2_^−^ / NO_3_^−^ as electron acceptors, the availability of competing electron acceptors such as O_2_, and the availability of suitable organic or other reduced substrates as electron donors. These factors may be impacted by the spatial proximity to linked processes such as NH_4_^+^ oxidation. Another process of N_2_ production is anaerobic NH_4_^+^ oxidation (anammox) with NO_2_^−^ as electron acceptor. Anammox occurs in marine and freshwater environments [14, 20, 21] and may be a particularly important N-loss process in oligotrophic lake sediments [22, 23]. The importance of DN and anammox relative to DNRA regulates N loss versus retention in an ecosystem. Hence, the balance of these three competing N-turnover processes, which often co-occur in freshwater lake sediments [23–26], represents an important constraint on the overall N budget of freshwater ecosystems.

Previous studies in aquatic environments have employed a variety of methods to identify and characterize N-transformation processes and/or the genes involved (e.g. [16, 23, 26, 27]). While ex-situ incubations with addition of (^15^N-labeled) substrates provide useful information on potential process rates [16], the experimental conditions deviate from those in-situ, and rates cannot be linked to the microbes involved in the process. Gradient-based rate modeling provides overall in-situ net rates, but does not allow us to study rates across smaller scales (e.g. sediment layers) [23]. On the other hand, functional gene quantification, sequencing, or metagenomics [23, 26, 27] provide only information on the potential of a community to perform certain pathways, but contain no information about the actual level of activity. Meta-transcriptomics, i.e. the profiling of whole-community RNA, has become an important way to assess in-situ functional activity [28]. Proteomics, can provide an additional level of information, i.e. the presence and abundance of enzymes involved in biochemical pathways [29], but was not used in this study. Quantification of actively transcribed functional traits was thus used here to link genetic potential to specific biogeochemical processes [18,30–35] and their ecosystem functions [31].

Here, we studied the microbial N cycle in sediments of 12 lakes in Switzerland covering a range of trophic states. We assessed the composition and abundance of the microbial population involved in N-cycling, as well as the functional potential from metagenomic analysis, and transcription and expression from metatranscriptomic analysis, of N-transformation genes in sediment. Furthermore, we evaluated whether the metagenomic and metatranscriptomic inventories of the sediments varied with more general lake characteristics, such as trophic state, geochemical properties, and morphology. We hypothesized that the abundance and transcriptional activities of microbial groups involved in N transformation are controlled by substrate availability (i.e., NO_3_^−^ and OM), and by extension by lake productivity and morphology. However, we demonstrate here that N-gene expression and transcription of single benthic N-cycling reactions is highly variable and largely decoupled from gene abundance and trophic state.

## Material and methods

### Study sites

The 12 lakes selected for this study comprise a wide range of morphologies, eutrophication histories, and present-day trophic states (Table 1). All experienced increases in anthropogenic P loading over the past century, which peaked in the 1970s. In the following decades, several of the lakes returned to meso- and oligotrophic conditions, while others (BAL, HAL, SEM) remained highly productive, and artificial hypolimnetic oxygenation was applied as a restoration strategy [36]. General limnological parameters and lake characteristics used for correlation analyses are provided in Table 1.

**Table 1:** Main morphological and trophic characteristics (based on P concentrations) of the studied lakes. Eu = Eutrophic, Meso = Mesotrophic, Oligo = Oligotrophic, AO = Artificially Oxygenated.

**Lake**	**Lake group**	**Surface area [km** ^**2**^ **]**	**Water residence time [yr]**	**Mean depth** **[m]**	**Maximum depth [m]**	**Trophic characteristics**
Brienz (BRI)	Pristine-alpine	29.8	2.69	168	260	Oligo
Lucerne (LUC)		111	3.5	107	214	Oligo
Sarnen (SAR)		7.15	0.76	34	52	Oligo
Walen (WAL)		24.1	1.45	104	145	Oligo
Baldegg (BAL)	Agriculture-influenced	5.22	4.22	33	66	Eu, AO
Hallwil (HAL)		9.95	3.85	29	45	Meso, AO
Sempach (SEM)		14.1	17.5	46	86	Meso
Zug (ZUG)		38.3	15.1	84	197	Eu
Constance (CON)	Large-deep	482	4.35	99	254	Oligo
Geneva (GEN)		582	11.4	153	310	Meso
Maggiore (MAG)		212	4.1	177	372	Oligo-meso
Neuchâtel (NEU)		213	8.25	64	153	Oligo-meso

### Sampling

Four sediment cores were collected between August and October 2019 from the deepest oxic region of each lake, avoiding sites known to be affected by landslides or nearby tributaries (Table S1). Oxygen profiles were recorded on one core with a vertical resolution of 100 μm using a PreSens O_2_ micro-optode (PreSens, Regensburg, Germany). 15 porewater samples were collected from a second core using MicroRhizons as described previously (0.25 cm intervals from 0–2 cm, 0.5 cm intervals from 2.5 to 5 cm; an additional water sample was obtained from water 1 cm above the core) [10, 23, 37]. Sediment material was extruded from the two remaining cores and sampled for bulk OM (0–5 cm; resolution: 0.5 cm) and microbiological (0–1 cm; resolution: 0.5 cm) analyses. For OM analysis, equal volumes of sediments from each core were collected and placed in pre-weighed plastic boxes to obtain ~5–20 g of sediment in total. Sediment for nucleic acid extraction was obtained with a clean, sterile, cut-off syringe (3 ml and 5 ml) and transferred into 2 ml DNA/RNAse free Eppendorf tubes, which were then flash-frozen with liquid N, and stored at −80°C until DNA/RNA extraction (each core and sediment depth resulted in five subsamples). Bulk OM samples were stored at −20°C prior to freeze-drying. Supplementary Table S2 provides an overview of the obtained samples.

### Sediment porewater and bulk chemistry

Porewater samples were analyzed by ion chromatography (882 IC Compact plus, Metrohm, Herisau, Switzerland); a 1:1 dilution with 2 mM HNO_3_ was required for cation determination, but not for anions. Freeze-dried samples were ground in an agate mortar. Total N and ^15^N/^14^N ratios (δ^15^N) were determined using an elemental analyzer (EA; Vario PYRO CUBE CN, Elementar, Langenselbold, Germany), connected to an isotope-ratio mass spectrometer (IRMS; Isoprime, Elementar). Sediment samples were acidified to remove carbonates, and subsequently analyzed for TOC and δ^13^C-TOC using the same EA-IRMS. Acetanilide (Schimmelmann, University of Indiana), Urea Working Standard (IVA Analysentechnik e. K.) and Boden Standard 1 (HEKAtech GmbH) were used as isotopic standards. The TP content of the sediments was determined photometrically after digestion with potassium peroxydisulfate in an autoclave for 2 h, using a Continuous Flow Analyzer (SAN++, Skalar, Breda, Netherlands).

### Data analysis and lake categorization

All graphs and statistical analyses were created in R (Version 3.6.3). We assigned the lakes to three groups based on ordination analyses (PCA, Fig. S1) of porewater electron acceptor and donor concentrations (O_2_, NO_3_^−^, SO_4_^2−^, NH_4_^+^) (Fig. S2A-B), bulk OM properties (TOC, TN, δ ^13^C-TOC, δ ^15^N-TN) (Fig. S2C), and information on location, morphology (Table 1) and land use. The three groups were agriculture-influenced lakes (BAL, HAL, SEM, ZUG; yellow shades), pristine-alpine lakes (BRI, LUC, SAR, WAL; red shades), and large-deep lakes (CON, GEN, MAG, NEU; gray shades). These parameters were considered as potential driving forces modulating the benthic N cycle.

### DNA and RNA extraction

RNA and DNA were extracted from sediment samples using the RNeasy PowerSoil Total RNA Kit (Qiagen, Hilden, Germany) and RNeasy PowerSoil DNA Elution Kit (Qiagen), following the manufacturer’s protocols. DNA yield was assessed using a NanoDrop One/One Microvolume UV–Vis Spectrophotometer (ThemoFisher Scientific, Wilmington, DE, USA). For each sediment depth of every lake, 3–9 samples were extracted separately; DNA and RNA samples of each lake and sediment sampling depth were subsequently pooled to provide enough DNA and RNA material for sequencing. Supplementary Table S3 provides an overview of the number of replicates pooled per DNA sample.

### 16S rRNA qPCR

Bacterial abundance was assessed using qPCR of 16S rRNA genes using primers Bact 349F and, Bact 806R and TaqMan probe Bact 516F_FAM [38] on a LightCycler 480 System (Roche, Basel, Switzerland). For details see *Supplementary methods, 16S rRNA qPCR*.

### Amplicon sequencing, pipeline, and bioinformatics

16S rRNA gene amplicon sequencing of the V3-V4 region was performed by Novogene (Hong Kong) with Illumina HiSeq technology to generate 2 × 300 bp paired-end reads following standard sequencing and quality control protocols. The sequences were analyzed separately for each lake using the DADA2 pipeline in R (version 3.5.1) [39]. For details see *Supplementary methods, Amplicon sequencing*.

The ASV abundance tables and the corresponding environmental parameters were further analyzed by calculating the beta diversity (ordination analysis), testing for significantly different microbial community composition between the lakes, locations, sampling months, and sediment sampling depth, using adonis. All results were visualized with the phyloseq, ggplot2, vegan, and microbiomeSeq packages using R statistical software (Version 3.6.3) [40–43]. The abundance table is available from the ERIC open data repository (see *Data availability*).

### Metagenomic and metatranscriptomic sequencing, pipeline, and bioinformatics

The metagenomes and metatranscriptomes were sequenced using Illumina NextSeq to generate 2 × 150 bp paired-end reads (averaging ~350 bp in length) by Novogene (Hong Kong), following rRNA depletion for metatranscriptomes and standard library generation and quality control protocols (see *Supplementary Methods* for details). Metagenomic and metatranscriptomic datasets were processed as previously described [44], and a detailed description is provided in *Supplementary methods, Metagenomics and metatranscriptomics*. Briefly, BBMap (v.38.71) was used to quality-control sequencing reads. Quality-controlled reads were merged using bbmerge.sh. Metagenomic reads were assembled into scaffolded contigs (hereafter scaffolds) using the SPAdes assembler (v3.15.2) [45]. Gene sequences were predicted using Prodigal (v2.6.3) [46]. Representative gene sequences were obtained by clustering at 95% identity, aligning against the KEGG database (release April.2022) using DIAMOND (v2.0.15) [47] and filtering (subject coverage of 70%; bitScore of at least 50% of the maximum expected bitScore).

The metagenomes were mapped to the reference genes and results were filtered to retain only alignments with a percentage identity of ≥95% and ≥45 bases aligned. Gene abundance profiles were calculated from best unique alignments and, for ambiguously mapped inserts, adding fractional counts to the respective target genes in proportion to their unique insert counts and dividing the total insert counts by the length of the respective gene. Functional abundance profiles were obtained by summing over shared KEGG Orthologies (i.e., same KO).

Gene-length normalized read abundances were further converted into per-cell gene and transcript copy numbers by dividing them by the median abundance of 10 single-copy marker genes copies [48] (MGs). The normalized metatranscriptomic abundance is given as the relative per-cell number of transcripts of a given functional group. Gene expression profiles, representing the relative number of transcripts per gene copy, were computed as the ratio between the metatranscriptomic profiles and metagenomic profiles [31].

The vegan package in R was used to analyze β-diversity between lakes from N-transformation genes and transcript abundances, and from the exponential of gene expression scores by ordination (metaMDS). Explanatory environmental parameters were obtained using envfit and bioenv. The co-expression was calculated using the network analysis from igraph (R package) based on Pearson correlation. To reduce the data noise, we only incorporated co-expression correlation with a *P*-value >0.8/≤0.8. All computations were performed based on log2-transformed values of the metagenomic and metatranscriptomic count profiles.

## Results

The 12 selected Swiss lakes represent a range of trophic states and areal N loads (Table 1). As a basis for further analysis, we assigned the lakes to three groups (agriculture-influenced, pristine-alpine and large-deep lakes) (see Methods—Data analysis and lake categorization, Table 1, Table S1). The grouping was firstly based on the ordination analyses of their geochemical properties such as sediment C, N, and P content, electron acceptor concentrations, and C/N isotopic signatures (Fig. S1). The grouping also took into account additional information on lake morphology and catchment land use, as will be discussed below.

### Porewater composition and benthic fluxes

**Figure 1:**
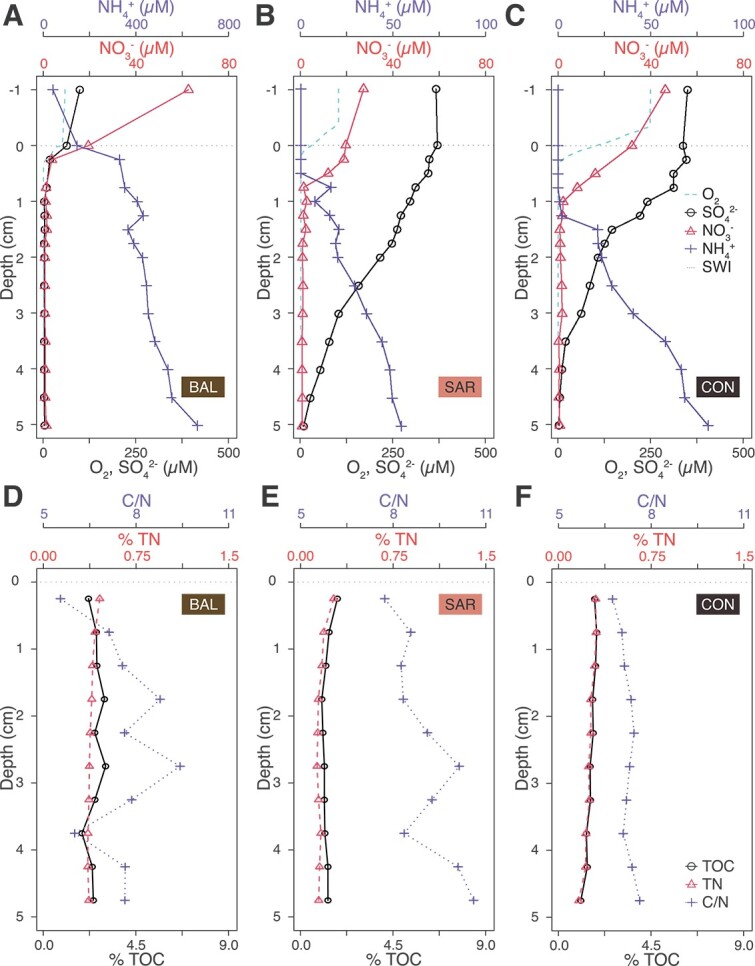
Representative profiles of porewater chemistry (A-C) showing O_2_ (dashed line), SO_4_^2−^ (open circles), NO_3_^−^ (open triangles) and NH_4_^+^ (crosses) concentrations (in μM) for an agriculture-influenced lake (BAL, A) a pristine-alpine lake (SAR, B) and a large-deep lake (CON, C). And profiles of bulk organic matter properties (D-F) showing TOC % (black lines), TN % (dashed lines) and molecular C/N ratio (dotted lines) as function of sediment depth for the same lakes. The horizontal dashed line marks the sediment–water interface (SWI). Note that the scale of NH_4_^+^ concentrations in the graph (a) differs from that of (b) and (c).

The sedimentary biogeochemistry and typical porewater redox concentration profiles were different for the three groups of lakes (representative examples (BAL, SAR, CON) in Fig. 1a-c; complete data set of porewater solute concentration profiles in Fig. S2a-b). While the sediments from all studied lakes were characterized by a shallow O_2_ penetration depth, lower O_2_ penetration depths were observed in the more eutrophic lakes (e.g., 1.2 mm in BAL versus 4.7 mm in WAL). Bottom water O_2_ concentrations ([O_2_]_bottom_) showed a strong correlation with water depth at the sampling site, as well as mean and hypolimnion depths (*r* = 0.8, 0.7, and 0.8, respectively, *P* < 0.05, Fig. S3), with shallow lakes (<100 m water depth) having lower [O_2_]_bottom_ (<110 μM), and deeper lakes (>100 m) having higher [O_2_]_bottom_ (>160 μM), regardless of the trophic state. This resulted in calculated O_2_ fluxes between 2.5 mmol m^−2^ d^−1^ in SAR to 10.7 mmol m^−2^ d^−1^ in GEN (Table 2).

**Table 2:** Benthic fluxes (in mmol m^−2^ d^−1^) of O_2_, NH_4_^+^, and NO_3_^−^ in the 12 studied Swiss lakes. Positive values represent fluxes from the overlying water into the sediments, negative values represent fluxes from the sediments to the overlying water.

**Lake**	**Lake Group**	**Flux [mmol m** ^**−2**^ **d** ^**−1**^ **]**		
		**O**_**2**_	**NH**_**4**_^**+**^	**NO**_**3**_^**−**^
BRI	Pristine-alpine	8.59	– 0.15	0.60
LUC		6.72	– 0.63	0.74
SAR		2.58	– 0.19	0.46
WAL		6.88	– 0.30	0.88
BAL	Agriculture-influenced	8.77	– 4.19	3.14
HAL		2.73	– 3.14	1.16
SEM		5.41	– 0.43	0.31
ZUG		7.34	– 2.03	0.21
CON	Large-deep	4.91	– 0.29	1.33
GEN		10.7	– 0.43	0.67
MAG		6.10	– 1.44	0.96
NEU		7.79	– 0.23	2.41

Net NO_3_^−^ flux into, and NH_4_^+^ flux out of, the sediments were determined from vertical porewater concentration gradients, and varied significantly among the considered lakes, ranging from 0.18 mmol m^−2^ d^−1^ in ZUG to 2.56 mmol m^−2^ d^−1^ in NEU for NO_3_^−^, and from −0.15 mmol m^−2^ d^−1^ in BRI to −4.19 mmol m^−2^ d^−1^ in BAL for NH_4_^+^ (Table 2). The O_2_ penetration depth correlated with the NH_4_^+^ flux (*r* = 0.7, *P* < 0.05, Fig. S3), but not with the NO_3_^−^ flux.

The concentration profiles of oxidants (NO_3_^−^ and SO_4_^2−^) and NH_4_^+^ displayed systematic differences between agriculture-influenced and pristine-alpine systems (Fig. 1a-b). In the former group, rapid depletion of all oxidants occurred mostly within the top 0.5 cm, and NH_4_^+^ accumulated rapidly with sediment depth, showing the steepest concentration gradient immediately below the oxycline, and exceeding 300 μM at 5 cm depth. In the pristine-alpine systems, NO_3_^−^ and SO_4_^2−^ penetrated much deeper, to >1 cm and ≥5 cm, respectively. NH_4_^+^ was below detection at the lower end of the oxycline, and increased below, yet at significantly lower concentrations than in agriculture-influenced lakes (Fig. 1b). Porewater profiles from large, deep (>100 m) lakes closely resembled those of pristine-alpine systems (low NH_4_^+^ accumulation, deeper NO_3_^−^ and SO_4_^2−^ penetration) (Fig. 1c).

### Bulk OM elemental and C and N stable isotopic composition

The OM export to the sediments varied among the three groups (exemplary data in Fig. 1d-f; complete data set on down-core bulk OM characteristics in Fig. S2c). The three lake groups differed in the sedimentary TOC and TN contents: while pristine-alpine lakes displayed the lowest TOC (<1.5%) and TN (<0.2%) contents, the highest TOC and TN contents (up to 9% and 1.25%, respectively) were observed in the agriculture-influenced and some of the deeper lakes (Fig. 1d-f; Fig. S4), indicating high OM reactivity. Both TOC and TN were significantly correlated with the total P concentrations in the top 15 m of the water column (*r* = 0.9 and 0.9, respectively, *P* < 0.05) (Fig. S3), confirming an influence of trophic state on OM export to the sediment. The average C/N ratio of ~8 was relatively close to the Redfield ratio (6.6, Fig. S2c), though higher values were observed in MAG and pristine-alpine lakes BRI, SAR, and WAL (depth-integrated C/N ratios between 8.5 and 9.5) (Fig. S2c). There were no clear downcore C/N ratio trends observable in any of the lakes (Fig. 1d-f, Fig. S4a), and TOC and TN contents strongly correlated with each other across all lakes and sediment depths (*r* = ~ 1, *P* < 0.05) (Fig. S3, S4a).

OM in pristine-alpine lakes was characterized by a relatively high δ^13^C (> −32‰, mostly −29.5‰ to −26‰) and a low δ^15^N (0‰ to +4‰), whereas the opposite held true for the OM in agriculture-influenced lakes (δ^13^C < −32‰ and δ^15^N > +4‰); in large-deep lakes, we observed mostly intermediate values (Fig. S4b). A systematic increase was observed for δ^13^C values with sediment sampling depth, whereas no clear trend was found for δ^15^N (Fig. S4b). The N isotopic composition of OM can help track the origin of the N that was incorporated, thanks to established source-specific isotopic ranges; for instance, N from synthetic fertilizers and manure have distinct isotopic signatures (δ^15^N ~ −3‰ to 3‰ and +10 to +20‰, respectively) [49].

### Microbial N transformation genes and their transcription

Metagenomes and -transcriptomes were obtained from the same sediment samples. This allowed us to assess not only the relative abundance of inorganic N-transformation genes (hereafter referred to as N-transformation potential), but also the relative abundance of the transcripts of these genes (referred to as N-gene transcription), as well as the N-gene expression (ratio of relative gene transcript abundance to relative gene abundance).

#### N-transformation genomic potential distinguishes two groups of lakes

**Figure 2:**
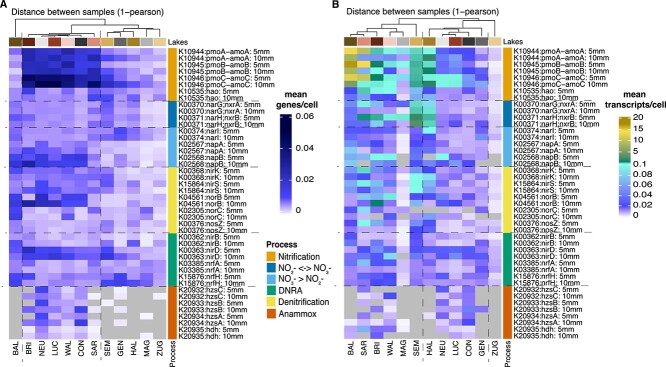
Heatmaps of N-transformation potential and transcription, i.e. the mean relative N gene abundance (genes/cell) (A) and mean N gene transcript abundance (transcripts/cell) (B) for the two sampled sediment depths of each lake. The lakes are colored (see upper x-axis color bar) according to the lake group colors from the physico-chemical definition (yellowish = agriculture-influenced lakes, reddish = pristine-alpine lakes, greyish = deep- large lakes, see also Fig. 3). The surface and deeper sediment samples are plotted per lake and gene (sample depth defined by 5 mm and 10 mm, respectively). The genes are colored according to N transformation process (see y-axis color bar). The genes of the NO_3_^−^ ↔ NO_2_^−^ process (dark blue) can be assigned either to NO_3_^−^ reductase (*narGH*) or NO_2_^−^ oxidation (*nxrAB*).

**Figure 3:**
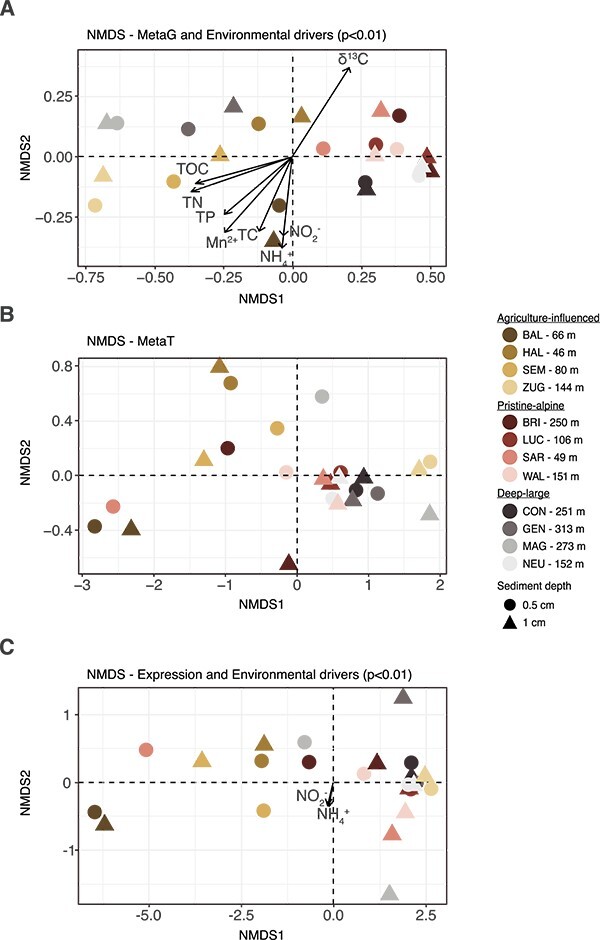
Non-metric multidimensional scaling ordination analysis based on the normalized abundances of inorganic N genes/cell (A) or their transcripts/cell (B). (C) NMDS of gene expression (log of the ratio of per cell abundance of genes and transcripts). The environmental parameters with the best fit to the ordination (significance level *P* < 0.01) are shown as vectors (length scaled: Arrow*0.5); there was no significant environmental parameter explaining the transcripts/cell. The samples are colored by the group and shaded by lake (yellow shades = agriculture-influenced lakes, red shades = pristine-alpine lakes, gray shades = deep-large lakes). The symbol indicates the sediment depth. The depth of the water column, where the sediment sample was taken, is indicated.

The abundance patterns of N-transformation potential of the lakes fell into two distinct groups, according to hierarchical cluster analysis (Fig. 2a) and ordination analysis (Fig. 3a). The highest N-transformation potential (>0.006 N-transformation genes/cell) was found in the pristine-alpine lakes and two large-deep lakes (NEU, CON), whereas MAG, GEN and the agriculture-influenced lakes displayed a lower N-transformation potential (<0.006 genes/cell) (Fig. 2, Dataset 1). This difference in N-transformation potential was driven by a higher potential for nitrification (0.017 genes/cell), DN (0.004 genes/cell), NO_3_^−^ reduction (0.005 genes/cell), and anammox (0.004 genes/cell) in the former compared to the latter six lakes (nitrification: 0.004 genes/cell, DN: 0.002 genes/cell; NO_3_^−^ reduction: 0.002 genes/cell, anammox: 0.00005 genes/cell) (Fig. 2a, Dataset 1).

The differences in N-transformation potential between the two different groups of lakes were generally greater than differences between the two sediment layers sampled within each core (0–5 mm and 5–10 mm). Nevertheless, some potential functions changed notably with sediment sampling depth—see e.g. *amoA* in SEM, GEN and HAL, anammox genes in NEU, or *napA*/*B* and *nirS*/*K* in BRI, LUC, WAL, CON, and SAR (Fig. 2a). Accordingly, ordination analysis of the β-diversity of the N-transformation potential revealed greater differences between lakes than between sediment sampling depths of the same lake (Fig. 3a). Agriculture-influenced lakes, together with lakes GEN and MAG were spread along axis 1 on the left half of the plot, with BAL separated along axis 2. The pristine-alpine lakes and deep lakes CON and NEU formed a tighter cluster. The deep lakes were all distinct and spread along axis 1. The TOC and TN contents correlated with axis 1 and best explained the different N-transformation potentials, with clear contrast between the lakes with high TOC and TN (e.g., GEN and MAG (deep-large) and ZUG and HAL (agriculture-influenced)) and those with low OM reactivity (low TOC and TN) on the other hand (e.g. pristine-alpine lakes).

#### N-gene transcription deviates from N-transformation potential

Across all lakes, the most highly transcribed N-transformation processes were nitrification (average 1.2 transcripts/cell), NO_2_^−^ oxidation/NO_3_^−^ reduction (average 0.1 transcripts/cell) and DN (average 0.04 transcripts/cell) (Fig. 2b, Dataset 2). Several genes for these functions were linked in a co-expression network (Fig. S5). Clustering based on N-gene transcript abundances produced distinct groupings from clusters based on N-gene potential (Fig. 2). One group contained the shallow lakes BAL, SEM, SAR, the two pristine-alpine lakes BRI and WAL and the deep-large lake MAG (Fig. 2b; Fig. 3b). These lakes showed more transcripts for nitrification and DN, especially in the surface sediments (Fig. 2b, Dataset 2). The other group encompassed the agriculture-influenced lakes ZUG and HAL, the deep-large lakes NEU, CON and GEN, and the pristine-alpine lake LUC, which displayed overall lower transcriptional activity (Fig. 2b). Differences between the sampled sediment layers were also more pronounced than for N-transformation genomic potential (Fig. 2b).

#### N gene expression highest in shallow lakes

**Figure 4:**
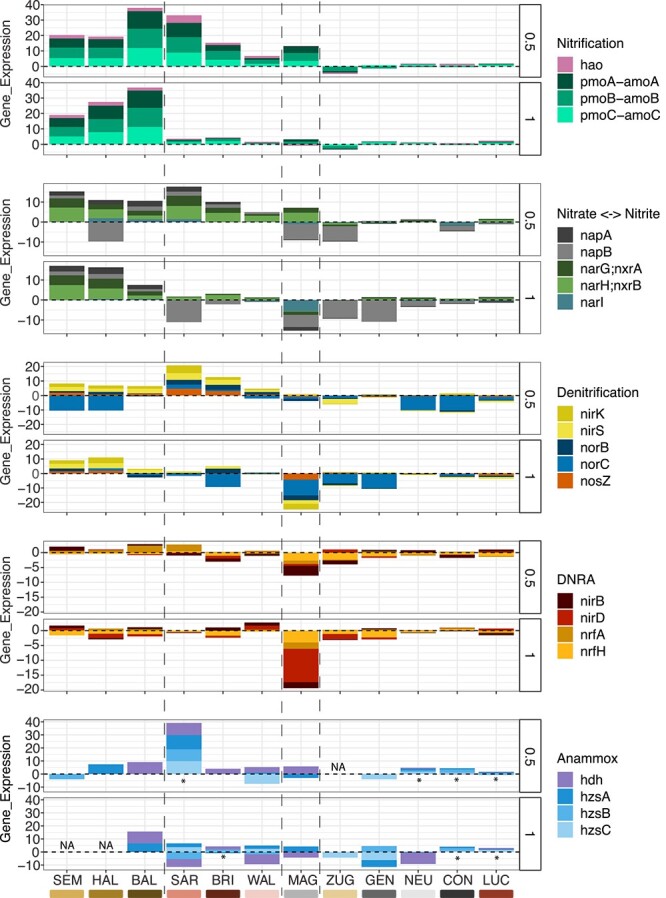
Gene expression for each of the targeted N-cycle processes, for different lakes and sediment depths (0.5 cm, 1 cm). Colors indicate genes. In the NO_3_^−^ ↔ NO_2_^−^ process, *narG*;*nxrA*, and *narH*;*nxrB* can be assigned either to NO_3_^−^ reduction (*narGH*) or NO_2_^−^ oxidation (*nxrAB*). The asterisk (*) indicates samples, in which the expression of all four genes of the anammox pathway was detected, ND = none detected).

For gene expression, defined as the ratio of gene and transcript abundance (Dataset 3), NMDS identified again a different grouping of lakes and samples (Fig. 3c, Dataset 3). In particular, we observed considerable differences between the sampled sediment depths within several lakes (especially MAG, SAR, HAL). In contrast, no obvious trends following the geochemically defined lake groupings were observed. In Fig. 4, lakes are therefore re-ordered according to patterns of gene expression observed across N-transformation functions and sampling depths.

Looking at individual N-gene expression pathways and genes, we observed a pattern of higher and vertically extended nitrification and NO_3_^−^ reduction gene expression in shallower, eutrophic lakes, and generally lower expression in the deep lakes. Similar expression patterns were notable in the shallow, agriculturally influenced lakes BAL, HAL, and SEM (Fig. 4, left side), with high expression of nitrification genes *amoABC, hao,* and of the nitrate reduction genes (*nap*, *nar*, and *nxr* genes) in both layers. Three oligotrophic lakes (SAR, BRI, WAL) displayed high nitrification and nitrate-reduction gene expression in the top sediment layer, but decreasing with increasing lake depth (Fig. 4, Dataset 3). Among these lakes, SAR stood out with a particularly high expression of anammox and DN genes. MAG (large-deep) was similar to the above-mentioned group of lakes, except that *napB* and *narI,* as well as several other genes involved in DN and DNRA showed lower expression levels. The remaining lake sediments (Fig. 4, right) displayed generally neutral or reduced expression levels in all functional categories. This group included CON, GEN, and NEU from the large-deep lakes group, but also the sediments from the fairly deep eutrophic ZUG and oligotrophic LUC (Fig. 4, Dataset 3).

## Discussion

### Chemical conditions in lake sediment only partially explain microbial N-cycling potential, transcription, and expression patterns

In this work we set out to establish links between genomic and transcriptomic information on microbial N-cycling and observed patterns in geochemical conditions of lakes and their sediments. We expected microbial communities and their N-cycling functions to be governed closely by the in-situ conditions. The porewater nutrient profiles obtained in this study are comparable to previously reported data [50] and clustered into three groups. The grouping reflects the importance not only of lake productivity, but also of water column mineralization in modulating benthic chemistry. While agriculture-influenced lakes are characterized by highly reactive sediments (i.e., strong DIN gradients), DIN profiles in the large-deep and pristine-alpine lakes suggest comparably low N fluxes, with weak NO_3_^−^ gradients and the absence of NH_4_^+^ right below the oxycline. This pattern has previously been explained by a higher contribution of anammox [50, 51] relative to DN. While the N gene analysis revealed how this grouping partially determined microbial communities and their N-cycling potential, no clear expression pattern emerged for the lake groups. A clear co-expression of nitrification, DN and NO_3_^−^/NO_2_^−^ transformation genes indicated the importance of internal N-cycling in lake sediments. Furthermore, the seemingly more pronounced dependence of N gene expression on sediment depth rather than on geochemical characteristics suggests that micro-scale differences in the sediments are more relevant for the expression and transcript abundance than whole-lake properties. In the following discussion we look at various aspects of our results in more detail.

### The genomic potential and expression of the N-loss processes DN and anammox

In agreement with previous geochemical findings [6], N-cycling genetic potential and gene expression data indicated that DN was the main fixed-N loss process in all lake sediments. On the other hand, the anammox potential was significant in some of the pristine-alpine and large-deep lakes, but was almost negligible in the agriculture-influenced lakes. Our data thus supports observations that anammox is an active and occasionally important N-loss process, particularly in sediments of nutrient-depleted lakes [50, 51]. However, while the gene abundance data seem to support this notion, our data on gene expression is somewhat contradictory regarding this hypothesis. That is, while transcripts for the full anammox process were found in several deep or nutrient-depleted lakes, i.e. NEU, CON, LUC, and SAR (Fig. 4), it was highly expressed only in the surface sediment of SAR. Even there, DN was simultaneously expressed as well, suggesting that anammox was not the only, and likely not the dominant N-loss process even in the oligotrophic lakes. However, anammox process rates were not directly determined in this study, and thus, we cannot confirm the relationship of transcriptional activity and actual N-turnover rates.

Low overall microbial abundance was detected in pristine-alpine lakes; amplicon sequencing further indicated a low abundance of *Planctomycetes* (the phylum containing all known anammox bacteria), compared to potential heterotrophic denitrifiers (Fig. S6). Since our abundance data was normalized by gene/transcript per cell, the anammox expression per unit volume, or mass, of sediment is probably considerably lower compared to the expression of DN, if the microbial biomass and key-player abundances are accounted for.

The ubiquitous importance of DN was further inferred from the high expression of *nosZ*, even in oxic sediments. Aerobic DN, previously described in permeable sediments, may also contribute to total N_2_ production [16, 17], as *napA*, an indicator for aerobic DN, was highly expressed [13, 52]. Its expression was co-localized with other DN and nitrification genes, corroborating the importance of aerobic DN, and suggesting a close coupling of aerobic DN and nitrification in the studied sediments (Fig. S5; see next section). However, further analyses involving the reconstruction of metagenome-assembled genomes and rate measurements are necessary to better disentangle the role of aerobic DN in lake sediments.

### Simultaneous expression of reductive and oxidative N-transformation processes

Most N-transformation processes were expressed across all of the studied lakes to some extent. Yet, coupled nitrification-DN stood out as the overarchingly dominating process, which appeared to be ubiquitous and highly expressed. Coupling of nitrification and DN has been widely reported in sediments from different aquatic ecosystems, including lake sediments [53], the North Sea [15], riverbed sediments [54], and mountain lake sediments [55]. Only marginal rates of coupled nitrification-DN were previously reported for sediment at the deepest point of BAL [56]. However, at that time the sediment surface was reportedly anoxic, in contrast to our observations (Fig. 1). Our study adds observations on the level of genomic potential and transcriptional activity that deepen our understanding of the coupling of N transformation processes in freshwater lake sediments.

We found a co-expression network of DN and nitrification genes (Fig. S5), but no clear co-expression of these two processes with the other N-cycle transformations. This indicates that NO_3_^−^ from nitrification (incl. comammox) actively supports NO_3_^−^ reduction to NO_2_^−^, the first step of DN. We observed consistent co-expression of nitrification and DN even in lake sediments where the availability of the substrates for nitrification (NH_4_^+^) and DN (NO_3_^−^) was low. Hence, our data imply that substrate limitation does not necessarily mute the expression of specific N-transforming pathways in these sediments. For example, high nitrification gene expression was found in the pristine-alpine and large-deep lake sediments, where freshly mineralized NH_4_^+^ was scarce and sometimes appeared in quantifiable amounts only at sampling depths greater than 1 cm below the sediment water interface (Fig. S2b). Similarly, high expression of the NO_3_^−^ reductase gene was observed in the lower sediments of BAL and HAL, despite NO_3_^−^ depletion in the top few millimeters (Fig. S2a). We therefore speculate that N-transformation processes (i.e., nitrification, DN, anammox), as well as release of NH_4_^+^ from OM, are tightly coupled and cryptic, i.e. fast turnover rates at times prevent the accumulation of measurable concentrations of substrates (NH_4_^+^, NO_3_^−^, and/or NO_2_^−^). A recent study on N transformations in riverbed sediments reported a tightly coupled nitrification-N loss cycle, where N loss was essentially independent of NO_3_^−^ or NO_2_^−^ porewater concentrations [54]. They attributed the disconnection of N-transformation rates and N-compound concentrations either to cryptic, undetectable N-cycle processes, or possibly to an unknown N-cycling pathway, or organism [54].

The cryptic N-cycle hypothesis requires further experimental verification and additional complementary observational methods for confirmation.

### OM modulates microbial N-cycling expression in lake sediments

DN rates based on measured porewater concentrations agreed well with previously published areal DN rates (Fig. S7, [8]) and also correlated with [NO_3_^−^]_bottom_, thus confirming NO_3_^−^ as the best predictor of DN rates. In contrast, the structure and expression of N-cycle genes were better predicted by variables linked to OM quantity and quality, which indicates a complex relationship between controls of community composition (Fig. 3a, Fig. S6b), gene expression (Fig. 3b, c), and rates (see *Supplementary Results and Discussion*, *Fig. S7*).

The quantity (i.e., TOC and TN content) and bulk isotopic composition of settled OM emerged as the key observable modulators of the structure of the benthic N-transformation potential in lacustrine sediments (Fig. 3A). The sedimentary OM was mostly composed of autochthonous material (C/N ratio ~8, δ^13^C < −32‰) [57] (Fig. S2c, Fig. S4b). δ^15^N-OM likewise varied between lakes (1 < δ^15^N < 10), likely as a consequence of variable N sources, including N from forest soils, synthetic fertilizers and manure (δ^15^N ~ −8‰ to 10‰, −3‰ to 3‰, and +10 to +20‰, respectively [49]). Accordingly, TOC and TN contents were likely regulated by areal TP (TP in the upper 15 m) via primary production. A greater contribution of allochthonous material was observed in pristine-alpine lakes (higher C/N ratios, low δ^15^N) (Fig. S2c). The importance of lake productivity in constraining the OM export to the sediments declines in large-deep lakes due to the combination of high O_2_ availability (sampling depth vs. [O_2_]_bottom_: *r* = 0.8, *P* < 0.05) and extended exposure time to O_2_, which favor mineralization of materials settling through deep water columns. Studying some of the same lakes, Steinsberger et al. concluded that in deep lakes (>100 m depth) easily degradable autochthonous material is largely mineralized during particle sinking before it reaches the sediment surface [50]. This depletion of reactive OM would explain the low N-gene transcription (Fig. 2) and N-gene expression in the large-deep and some of the pristine-alpine lakes (Fig. 4). Indeed, the quantity, bulk isotopic composition, and C/N ratio of OM reaching the sediments notably differs among the 12 lakes (elemental C/N ratios ~5 to ~13 for surface sediments) (Fig. S2c). We can therefore assume a variable reactivity, i.e. potential of the deposited OM to undergo further sedimentary aerobic and anaerobic OM mineralization processes, with obvious implications for the release of NH_4_^+^ fueling the benthic N cycle and driving heterotrophic microbial activity, including DN.

The export of fresh, labile OM to the sediments is crucial for the N-cycling community. On the one hand, it provides bioavailable N compounds, such as NH_4_^+^ and NO_2_^−^ via OM mineralization, while on the other hand, it acts as an electron donor for heterotrophic N-cycle processes, such as organotrophic DN and DNRA [58, 59]. Thus, reduced availability of fresh OM provides a viable explanation for the low N-gene transcription and expression in the large-deep and pristine-alpine lakes compared to the shallower agriculture-influenced lakes BAL, HAL, and SEM, which are OM-rich (Fig. S4). Interestingly, the N-transformation potential was higher in pristine-alpine and some large-deep lakes, indicating that N transformation, even at this low level of transcription, is a successful strategy to gain energy in such N- and C-depleted sediments.

Two pristine-alpine lakes (SAR and BRI) diverged from this pattern and displayed a highly transcriptionally active N-cycling community in the surface sediment, and a transcription pattern more similar to agriculture-influenced lakes (Fig. 2b). The divergent behavior of these lakes could be influenced by the distinct allochthonous matter inputs from the catchment. Intense precipitation events trigger recurrent flooding of the tributaries to SAR [23, 60], leading to substantial inputs of allochthonous carbon. The high C/N ratio in the surface sediments of BRI supports substantial allochthonous OM input; however, in SAR this elemental ratio was low (Fig. S2C).

In summary, our analysis provides putative evidence that the abundance and activity of the N-cycling communities in Swiss lake sediments is driven by OM input and/or OM degradation in the water column, and not necessarily by the readily available inorganic N forms, such as NO_3_^−^ or NH_4_^+^. Supporting this notion, a drastic change in the transcription of N-cycling genes due to OM availability has been documented by transcriptomic data in a soil study [61]. However, the divergence of transcript abundance and gene expression patterns from N-transformation potential, and the lack of correlation with potential environmental drivers, indicates that transcriptional regulation in these systems is more complex, and requires further investigation.

### Differential controls on N-transformation genomic potential, transcription, and expression

Although sorting lakes into three groups appeared reasonable based on their similar geochemical characteristics, microbial data clearly depicted a more nuanced picture, where lakes within the same group displayed differences in their microbial N gene composition and transcriptional activity. Another key observation of this study is that the structure and relative abundance of the N-transformation genomic potential, the population-wide transcriptional activity of these functions, and the relative transcriptional activity per gene (expression) differ considerably, and thus appear to be differentially controlled in micro-scale niches. These differences could also arise from temporal decoupling, e.g., short term responses to current conditions reflected in transcriptional activity versus longer-term controls acting on the composition of the microbial communities involved.

While these results emphasize the value of using a multi-omics approach to study microbial transformations at these various levels, they also call for caution when attempting to establish simple relationships between any of these measures and N-cycle process rates. The combined metagenomic-metatranscriptomic approach used in this study thus provides valuable insights into the multi-layered controls on the microbial machinery underpinning biogeochemical processes (population size and structure, community-level transcriptional activity, gene-level expression), but cannot replace, or naively explain, process rate measurements.

## Conclusion

With this omics-driven survey of the microbial N cycle in the sediments of 12 Swiss lakes, we demonstrate that the N-gene transformation potential and gene expression of microbial communities exhibit considerable, and partly systematic, differences in response to limno-sedimentological and biogeochemical properties of these lakes.

Gene abundance and expression data indicate that DN, possibly including aerobic DN, is likely the main N-loss pathway performed by the studied sediment microbial communities. Yet, anammox genes are present and expressed, particularly in pristine-alpine and some large-deep lakes, indicating that anammox plays an important role for N loss from these lakes. Often, N processes were co-expressed (in particular nitrification and DN), even where the required DIN substrates were not detectable. This suggests an internal N cycle, with a rapid coupling of oxidative and reductive N-cycling reactions that prevent the accumulation of measurable concentrations of dissolved DIN. The canonically assumed links between N substrate availability, N-cycling process rates, abundance of N-cycling microorganisms, and the expression of N-cycling genes may thus be obscured. The different abundance structures and drivers we observed for functional potential versus community-level transcription and expression may further indicate the importance of microniches or of environmental controls acting on them on different temporal scales. While the environmental drivers of the composition and activity of N-cycling microbial communities could not be fully resolved, statistical analyses suggest OM quality/quantity as their main predictor. A better understanding of the role of OM in the regulation of N-cycle processes may thus be helpful when estimating DN/N loss under changing environmental conditions and sedimentation regimes.

## Supplementary Material

SwissLakes_2024_R2_Supplement_20240902_ycae110

SwissLakes_2024_SI-Datasets_R1_20240823_ycae110

## Data Availability

All sequences are available under NCBI BioProject PRJNA833462. All other data, such as metadata and the abundance tables, are available at the institutional research data repository of Eawag (ERIC) (https://opendata.eawag.ch/) in accordance with FAIR data sharing principles (10.25678/0009F7).
