# Supplementary material for: Metagenomic and -transcriptomic analyses of microbial nitrogen transformation potential, and gene expression in Swiss lake sediments
Source: ISME Commun. 2024 Oct 15;4(1):ycae110. doi: 10.1093/ismeco/ycae110 (PMC11476906; doi:10.1093/ismeco/ycae110)
Supplement: SwissLakes_2024_R2_Supplement_20240902_ycae110 [file swisslakes_2024_r2_supplement_20240902_ycae110.pdf]

Supplementary information for:

Metagenomic and -transcriptomic analyses of microbial nitrogen transformation potential, and gene expression in Swiss lake sediments

Kathrin B.L. Baumann*[1], Alessandra Mazzoli*[2], Guillem Salazar[3], Hans-Joachim Ruscheweyh[3], Beat Müller[1], Robert Niederdorfer[1], Shinichi Sunagawa[3], Mark A. Lever[4,5], Moritz F. Lehmann[2], Helmut Bürgmann[1,†]

[1] Eawag, Swiss Federal Institute of Aquatic Science and Technology, 6047 Kastanienbaum, Switzerland

[2] Department of Environmental Sciences, University of Basel, 4056 Basel, Switzerland

[3] Department of Biology, Institute of Microbiology and Swiss Institute of Bioinformatics, ETH Zurich, Zurich, Switzerland.

[4] Institute of Biogeochemistry and Pollutant Dynamics, ETH-Zurich, Zurich, Switzerland

[5] Now at Marine Science Institute, University of Texas at Austin, Port Aransas, TX, USA

* These authors contributed equally to the work

† Corresponding author

Supplementary methods

16S rRNA qPCR

Bacterial abundance was assessed using qPCR of 16S rRNA genes. The reaction mixture (10 µl total volume) included: 1 x Roche TaqMan Master Mix, 0.9 µM of each primer (Bact 349F, Bact 806R), 0.3 µM TaqMan probe (Bact 516F_FAM), molecular grade water (Qiagen) and 2 µl of DNA extracts or standards [1]. The employed thermal cycling was: 10 min at 95°C (initial denaturation; ramp time: 4.4°C/min) and 45 cycles of denaturation (40 s at 95°C), annealing (40 s at 53°C) and elongation (1 min at 72°C). The amplification was performed in technical triplicates using a LightCycler 480 System (Roche, Basel, Switzerland) in 384 well plates, and the results were normalized to the dry mass of extracted sediment. External standards were prepared from *E. coli* using pGEM T Easy (Promega, Madison, WI, USA) containing a 16S rRNA gene insert of 1465 bp length. Negative controls (reaction mixture + molecular grade water) and extraction blanks were included. Amplification efficiency (99%) and error of the external standard curve (R^2 = 0.999) were checked.

Amplicon sequencing

16S rRNA gene amplicon sequencing was performed by Novogene (Hong Kong) following standard sequencing and quality control protocols. The 24 DNA samples were amplified with universal 16S rRNA gene primers 341F (5'-CCT AYG GGR BGC ASC AG-3') and 806R (5'-GGA CTA CNN GGG TAT CTA AT-3') targeting the V3-V4 region (Fragment length ~466 bp), followed by size selection, end repair and A-tailing and adaptor ligation steps. The libraries were checked with Qubit and real-time PCR for quantification and bioanalyzer for size distributions and sequenced with Illumina HiSeq technology to generate 2x300 bp paired-end reads. This resulted in 24 samples with an average of 112,857 raw paired-end reads. The sequences were analyzed separately for each lake using the DADA2 pipeline in R (version 3.5.1). Briefly, after a quality check, the raw sequence adapters were trimmed, dereplicated and error rates were calculated before applying the DADA2 core sample inference algorithm. Next, the forward and reverse reads were merged to obtain amplicon sequence variants

(ASVs). Following chimera removal with the removeBimeraDenovo algorithm, the taxonomic assignment of the ASVs was performed using a Naïve Bayesian classifier method based on the SILVA database 138 for the V3/V4 region [2]. Unclassified ASVs and ASVs assigned to chloroplasts were filtered out. The ASV abundance tables and the corresponding environmental parameters were further analyzed by calculating the beta diversity (ordination analysis), testing for significantly different microbial community composition between the lakes, locations, sampling months, and sediment sampling depth, using adonis. All results were visualized with the phyloseq, ggplot2, vegan and microbiomeSeq packages using R statistical software (Version 3.6.3) [3–6]. The abundance table is available from the ERIC open data repository (see data availability).

Metagenomics and metatranscriptomics

The metagenomes and metatranscriptomes were sequenced using the Illumina NextSeq platform to generate 2 × 150 bp paired-end reads by Novogene (Hong Kong) following standard library generation protocols. Briefly, for metatranscriptomics, the RNA extracts were depleted for rRNA, followed by fragmentation, reverse transcription and second strand cDNA synthesis, end-repair and A-tailing, adaptor ligation, size selection and PCR amplification. The library was checked with Qubit and real-time PCR for quantification and bioanalyzer for size distribution. For shotgun metagenomics the same steps were followed for library generation omitting the RT and cDNA synthesis steps. Metagenomic and metatranscriptomic datasets were processed as previously described [7,8]. Briefly, BBMap (v.38.71) was used to quality control sequencing reads from all samples by removing adapters from the reads, removing reads that mapped to quality control sequences (PhiX genome) and discarding low-quality reads (*trimq=14, maq=20, maxns=1, and minlength=45)*. Quality-controlled reads were merged using bbmerge.sh with a minimum overlap of 16 bases, resulting in merged, unmerged paired, and single reads, each of which were considered as one ‘insert’ for gene profiling steps (see below). From transcriptomes, rRNA sequences were removed with sortmeRNA [9].

The reads from metagenomic samples were assembled into scaffolded contigs (hereafter scaffolds) using the SPAdes assembler (v3.15.2) [10] in metagenomic mode. Scaffolds were length-filtered (≥ 500 bp) and gene sequences were predicted using Prodigal (v2.6.3) [11] with the parameters *-c -q -m -p meta*.

Genes were subsequently clustered at 95% identity, keeping the longest sequence as representative using CD-HIT (v4.8.1) with the parameters *-c 0.95 -M 0 -G 0 -aS 0.9 -g 1 -r 0 -d*. Representative gene sequences were aligned against the KEGG database (release April.2022) using DIAMOND (v2.0.15) [12] and filtered to have a minimum query and subject coverage of 70% and requiring a bitScore of at least 50% of the maximum expected bitScore (reference against itself).

Metagenomes and transcriptomes were then mapped to the cluster representatives (i.e., reference genes) with BWA (v0.7.17-r1188; *-a*) [13] and the resulting BAM files were filtered to retain only alignments with a percentage identity of ≥95% and ≥45 bases aligned. Gene abundance profiles were calculated by first counting inserts from best unique alignments and then, for ambiguously mapped inserts, adding fractional counts to the respective target genes in proportion to their unique insert counts and dividing the total insert counts by the length of the respective gene. We subsequently converted the gene abundance profiles into functional abundance profiles by taking the sum of the length-normalized abundances across reference genes belonging to the same functional group based on shared KEGG Orthology (i.e., same KO).

Gene-length normalized read abundances were further converted into per-cell gene and transcript copy numbers by dividing them by the median abundance of 10 single-copy marker genes [14] (MGs) in each sample. MGs were selected as all genes with KEGG annotation corresponding to K06942, K01889, K01887, K01875, K01883, K01869, K01873, K01409, K03106, and K03110. MGs are particularly suitable for normalizing metagenomic and metatranscriptomic data to provide estimates of relative per-cell gene copies, because they

represent universal, single-copy and constitutively expressed housekeeping genes [8,15]. The normalized metagenomic abundance can therefore be interpreted as the per-cell number of gene copies of a given gene. Accordingly, the normalized metatranscriptomic abundance can be interpreted as the relative per-cell number of transcripts of a given functional group. Gene expression profiles, representing the relative number of transcripts per gene copy, were computed as the ratio between the log2-transformed, DESeq2-variance stabilized [16] and MG-median centered metatranscriptomic profiles (reflecting the relative number of transcripts per cell) and the metagenomic profiles (reflecting the number of gene copies per cell) [8].

The vegan package in R was used to calculate the distance (metaMDS) of N-transformation genes and transcripts between lakes, and to find explanatory environmental parameters (envfit and bioenv). The co-expression was calculated using the network analysis from igraph (R package) based on Pearson correlation. To reduce the data noisiness, we only incorporated co-expression correlation with a P-value >0.8/<-0.8. All computations were performed based on log2-transformed values of the metagenomic and metatranscriptomic count profiles.

Supplementary results and discussion

Divergence between microscale microbial processes and whole-lake budgets

In this supplementary discussion, we explore links between metagenomic and transcriptomic data and whole lake N removal rates.

Investigating the N cycle and N-transformation genes at a small scale (i.e., in a single core) enabled us to verify the role of OM properties in driving individual benthic microbiological processes. Metatranscriptomics highlighted the complexity of the relationship of genomic potential versus activity of the microbial N cycle in freshwater sediments at this scale. We showed in earlier work that significant spatial differences can occur in the N-transformation potential of sediments of a single lake, depending on sampling depth and associated spatio-temporal changes in the environment (e.g., redox regime, OM export flux) [17]. We therefore

want to discuss the extent, to which the analyses of the sediment cores investigated here, were representative of the lake systems studied.

Whole-lake budgets can be used to quantify the overall N-removal efficiency of lakes [18,19], based on the mass balance of N loading, export, and loss (mostly DN and burial) [20]. The fluxes calculated here from porewater NO_3^- profiles compare relatively well with trends in areal DN rates calculated by Müller et al (2022) for the same lacustrine systems (Figure S7A). Relative differences between lake groups are equally well reflected by the two approaches. In addition, both approaches agreed in that they similarly predict that bottom water NO_3^- concentration is the main driving factor modulating NO_3^- flux (Figure. S7B, [21]). Overall, the flux estimates from single cores seem representative for whole lakes, in spite of the disparity of the methods. However, in terms of absolute flux values, porewater-based flux estimates were ~40% lower than the estimates from whole lake budgets [19]. The difference between the two estimates was previously ascribed to processes occurring elsewhere in the lake (e.g. in the water column), as opposed to at the SWI. This discrepancy could also derive from uncertainty in the mass balance terms, insufficient resolution in the porewater concentration profiles, and/or local sediment heterogeneity. Moreover, Müller et al (2022) employed long-term datasets to generate annual DN rates, whereas our porewtare-concentration based fluxes, as well as our gene expression data represent snapshots in time, during the stratification phase, when NO_3^- consumption decays [21] and DN rates tend to decrease [22].

Thus our results are consistent with Müller et al 2022 in indicating that $[NO_3^-]_{bottom}$ is the main predictor of DN rates in the considered lakes based on whole-lake N budgets. However, the present study showed that the structure and expression of microbial N-cycle genes is primarily driven by bulk OM properties, and to a limited extent by NH_4^+, while $[NO_3^-]_{bottom}$ plays only a subordinate role. This suggests that the link between microbial N-community structure, transcriptional regulation, activity and process rates in benthic ecosystems is complex, and predictions of ecosystem functioning solely based on metagenomic/-transcriptomic analyses

are difficult. These results should be confirmed by other studies and methods, in particular linking transcriptional observations directly to rate measurements at the same scale.

Supplementary tables

Table S1: *Sampling point coordinates (CH1903+ LV95), depth and date of sampling. Lakes marked with * were not sampled at the deepest location*

Lake Group	Lake	Sampling Coordinates (CH1903+ LV95)		Sampling depth [m]	Data of sampling
		X	Y		
Pristine-alpine lakes	Brienz (BRI)	639226	174179	250	29/08/19
	Lucerne (LUC)*	669359	208244	106	26/08/19
	Sarnen (SAR)	658769	191107	49	04/09/19
	Walen (WAL)	735380	220704	151	26/09/19
Agriculture-influenced lakes	Baldegg (BAL)	662366	227683	66	02/09/19
	Hallwil (HAL)	658862	236313	46	17/09/19
	Sempach (SEM)	654659	221318	80	30/09/19
	Zug (ZUG)*	679543	219302	144	11/09/19
Large-deep lakes	Constance (CON)	749287	274886	251	24/09/19
	Geneva (GEN)	534721	144984	313	01/10/19
	Maggiore (MAG)*	698758	106354	273	19/09/19
	Neuchâtel (NEU)	554566	195117	152	09/09/19

Table S2: *Overview of samples/measurements taken and analyses performed, with number or replicate samples. Shaded cells indicate no sample obtained.*

<table>
<tr><th>Cores</th><th>Core I</th><th>Core II</th><th colspan="2">Cores III & IV</th></tr>
<tr><td>Sampling</td><td>None</td><td>Porewater sampling</td><td>Sediment sampling for bulk OM</td><td>Sediment sampling for microbiology</td></tr>
<tr><td>Methods</td><td>O_2 microprofiling</td><td>Ion chromatography</td><td>Elemental analyzer, isotope ratio mass spectrometer</td><td>Nucleic acid extraction (DNA & RNA)</td></tr>
<tr><td>Parameters</td><td>O_2</td><td>NH_4^+, Mn^{2+}, NO_3^-, SO_4^{2-} (Na^+, K^+, Ca^{2+}, Mg^{2+}, Cl^-, NO_2^-, PO_4^{3-})[a]</td><td>TOC, TN, ^{13}C-TOC, ^{15}N-TN isotopes</td><td>DNA -> Metagenomics, 16S rRNA gene amplicon sequencing RNA -> Metatranscriptomics</td></tr>
<tr><td>Depth</td><td>Measurements</td><td>Samples</td><td>Samples</td><td>Samples</td></tr>
<tr><td>-1-0cm</td><td></td><td>×1</td><td></td><td></td></tr>
<tr><td>0-0.25 cm</td><td rowspan="14">×1 (continuous microprofiling at 100 µm resolution)</td><td>×1</td><td rowspan="2">×1 (composite III&IV)</td><td rowspan="2">×5(III) ×5(IV)</td></tr>
<tr><td>0.25-0.5 cm</td><td>×1</td></tr>
<tr><td>0.5-0.75 cm</td><td>×1</td><td rowspan="2">×1 (composite III&IV)</td><td rowspan="2">×5(III) ×5(IV)</td></tr>
<tr><td>0.75-1 cm</td><td>×1</td></tr>
<tr><td>1-1.25 cm</td><td>×1</td><td rowspan="2">×1 (composite III&IV)</td><td rowspan="10"></td></tr>
<tr><td>1.25-1.5 cm</td><td>×1</td></tr>
<tr><td>1.5-1.75 cm</td><td>×1</td><td rowspan="2">×1 (composite III&IV)</td></tr>
<tr><td>1.75-2 cm</td><td>×1</td></tr>
<tr><td>2-2.5 cm</td><td>×1</td><td rowspan="2">×1 (composite III&IV)</td></tr>
<tr><td>2.5-3 cm</td><td>×1</td></tr>
<tr><td>3 – 3.5 cm</td><td>×1</td><td rowspan="2">×1 (composite III&IV)</td></tr>
<tr><td>3.5-4 cm</td><td>×1</td></tr>
<tr><td>4-4.5 cm</td><td>×1</td><td rowspan="2">×1 (composite III&IV)</td></tr>
<tr><td>4.5-5 cm</td><td>×1</td></tr>
</table>

[a] *Data not included in manuscript, available from the data repository.*

Table S3: *Overview of replicate samples used for nucleic acid (DNA and RNA) extraction for each lake and sediment depth. From each lake, two cores were sampled with 5 replicates per core and sediment depth for a total of 10 possible extractions (see Table S2). The number of extracted replicates depended on the DNA and RNA yield. Samples were chosen randomly for extraction, but at least one replicate per core was chosen. The replicate DNA and RNA extracts were subsequently pooled to yield one extract per lake and depth that was used for all downstream analyses (pooled DNA: qPCR, 16S rRNA gene amplicon sequencing, shotgun metagenomics. Pooled RNA: metatranscriptomics).*

**Lake**	**Sediment depth (cm)**	**Numbers of replicate samples used for extraction**
Baldegg	0.5	3
Baldegg	1	3
Hallwil	0.5	3
Hallwil	1	3
Sempach	0.5	3
Sempach	1	2
Zug	0.5	3
Zug	1	3
Neuenburg	0.5	2
Neuenburg	1	2
Geneva	0.5	3
Geneva	1	2
Maggiore	0.5	3
Maggiore	1	3
Lucerne	0.5	6
Lucerne	1	6
Sarnen	0.5	3
Sarnen	1	9
Walen	0.5	3
Walen	1	3
Brienz	0.5	6
Brienz	1	9

Supplementary figures

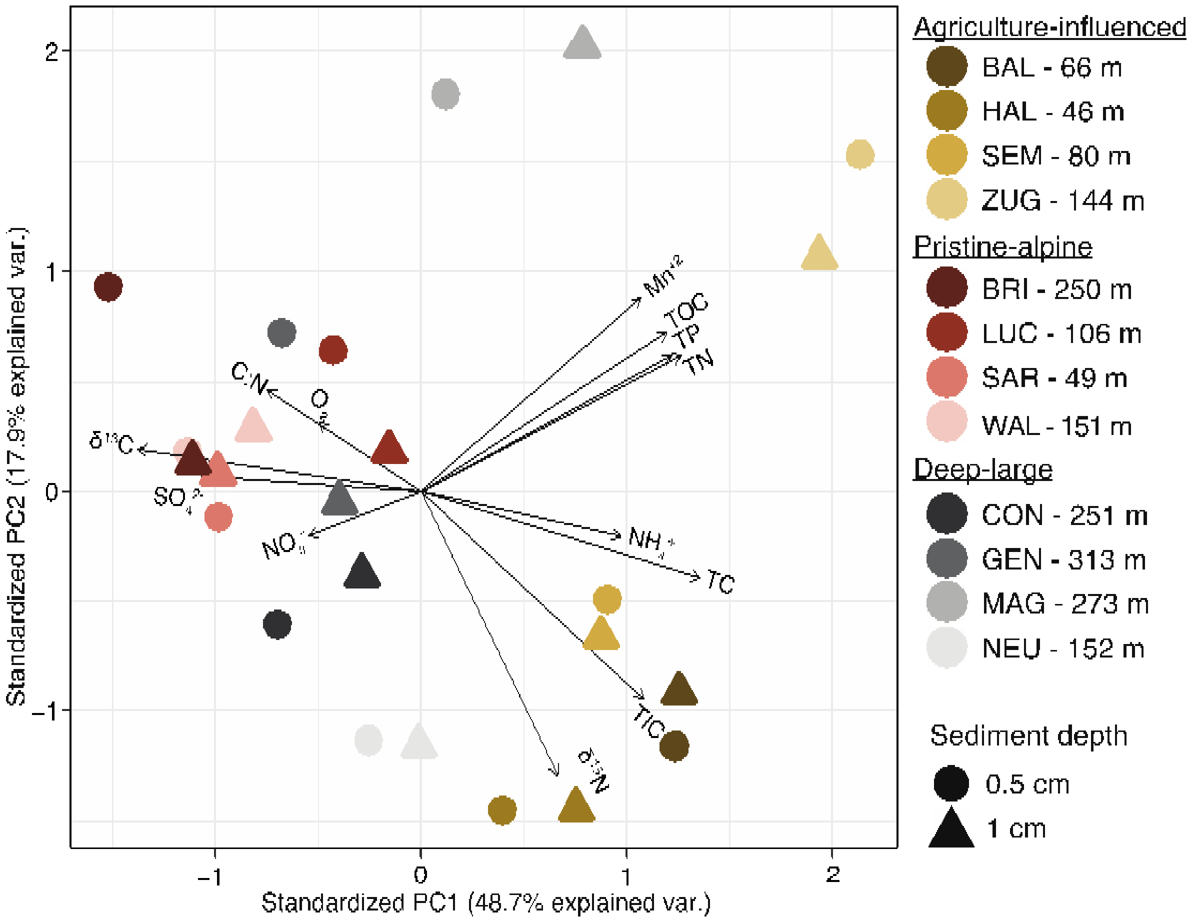

Figure S1: *Principal Component Analysis of the geochemical data of surface sediment (0 for porewater and 0-0.5 cm for bulk organic matter). The lakes are colored according to the group colors from the physico-chemical definition (yellowish = agriculture-influenced lakes, reddish = pristine-alpine lakes, greyish = deep- large lakes). Sampling depths for the respective lakes are indicated.*

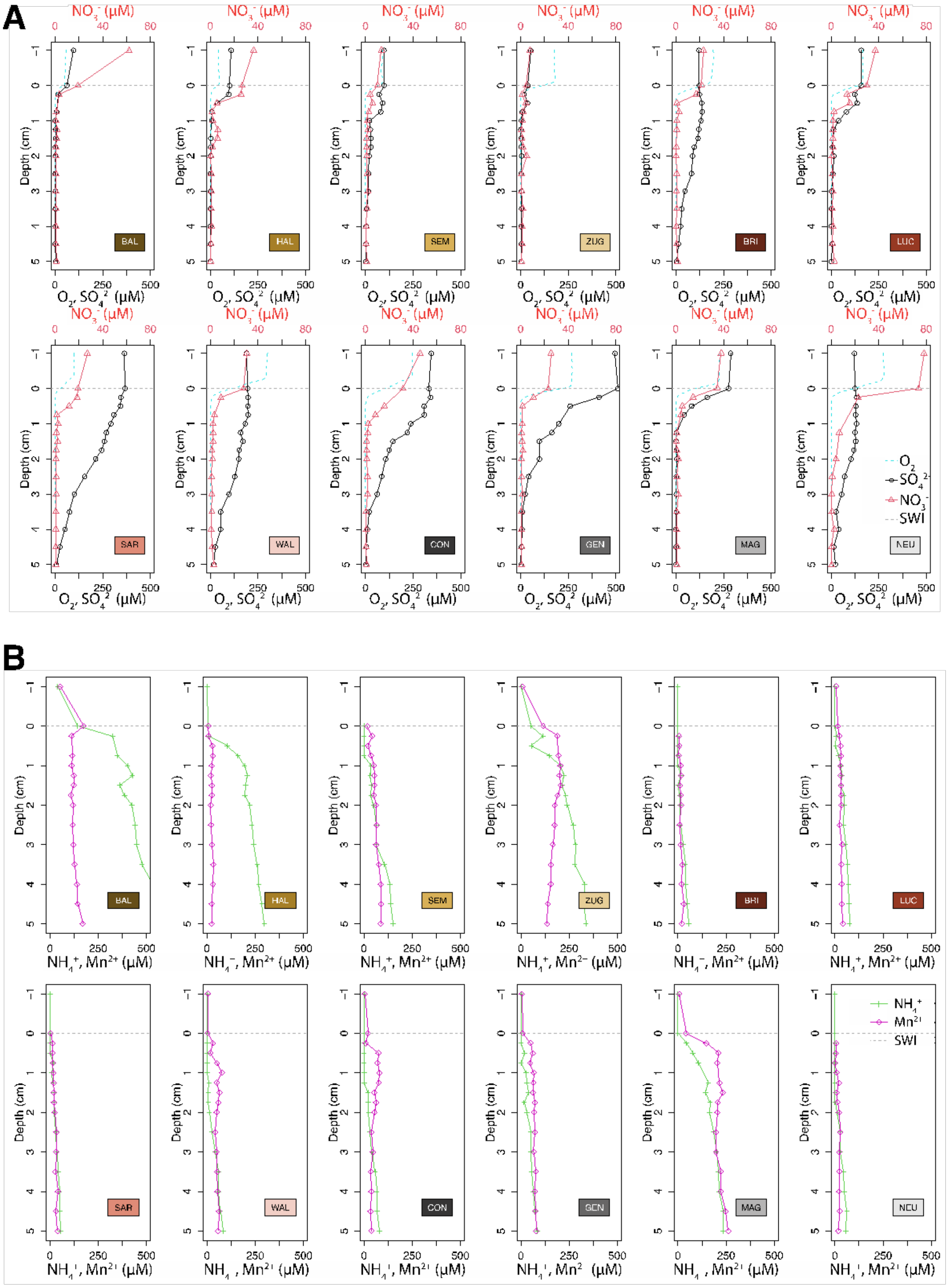

A
NO_3^- (µM)
O_2, SO_4^{2-} (µM)
Depth (cm)
BAL
HAL
SEM
ZUG
BRI
LUC
SAR
WAL
CON
GEN
MAG
NEU
O_2
SO_4^{2-}
NO_3^-
SWI
B
NH_4^+, Mn^{2+} (µM)
Depth (cm)
BAL
HAL
SEM
ZUG
BRI
LUC
SAR
WAL
CON
GEN
MAG
NEU
NH_4^+
Mn^{2+}
SWI

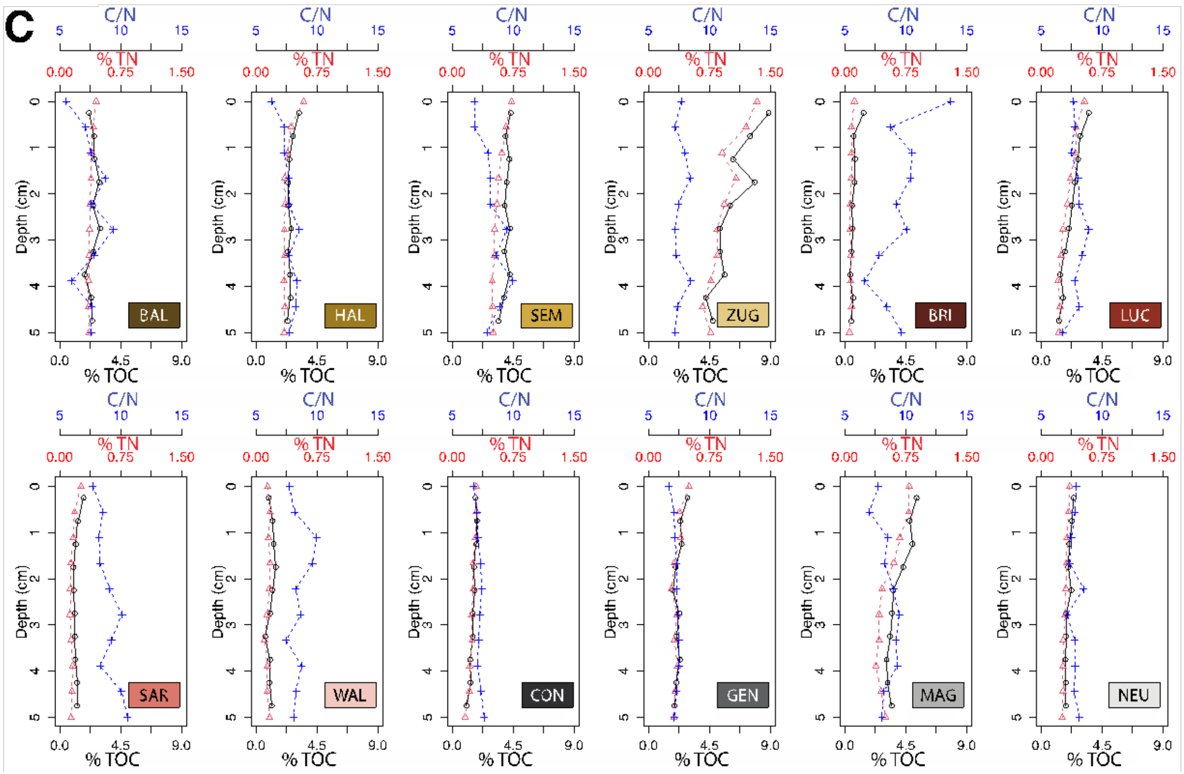

Figure S2: *Porewater concentration profiles of the (A) oxidants O_2 (light blue dashed line), SO_4^{2-} (black open circle) and NO_3^- (red open triangle) and (B) reductants NH_4^+ (green cross) and Mn^{2+} (pink open square). All concentrations are reported in µM; concentration data of reductants below the LOD were omitted. Sediment profiles of (C) bulk organic matter properties: elemental C/N ratio (blue line), TN (red line) and TOC (black line) contents (in %). Data from all twelve lakes are reported: Baldegg (BAL), Brienz (BRI), Constance (CON), Geneva (GEN), Hallwil (HAL), Lucerne (LUC), Maggiore (MAG), Neuchâtel (NEU), Sarnen (SAR), Sempach (SEM), Walen (WAL) and Zug (ZUG). The lakes are clustered by group according to the following color shading: yellow shades = agriculture-influenced lakes, red shades = pristine-alpine lakes, gray shades = deep-large lakes.*

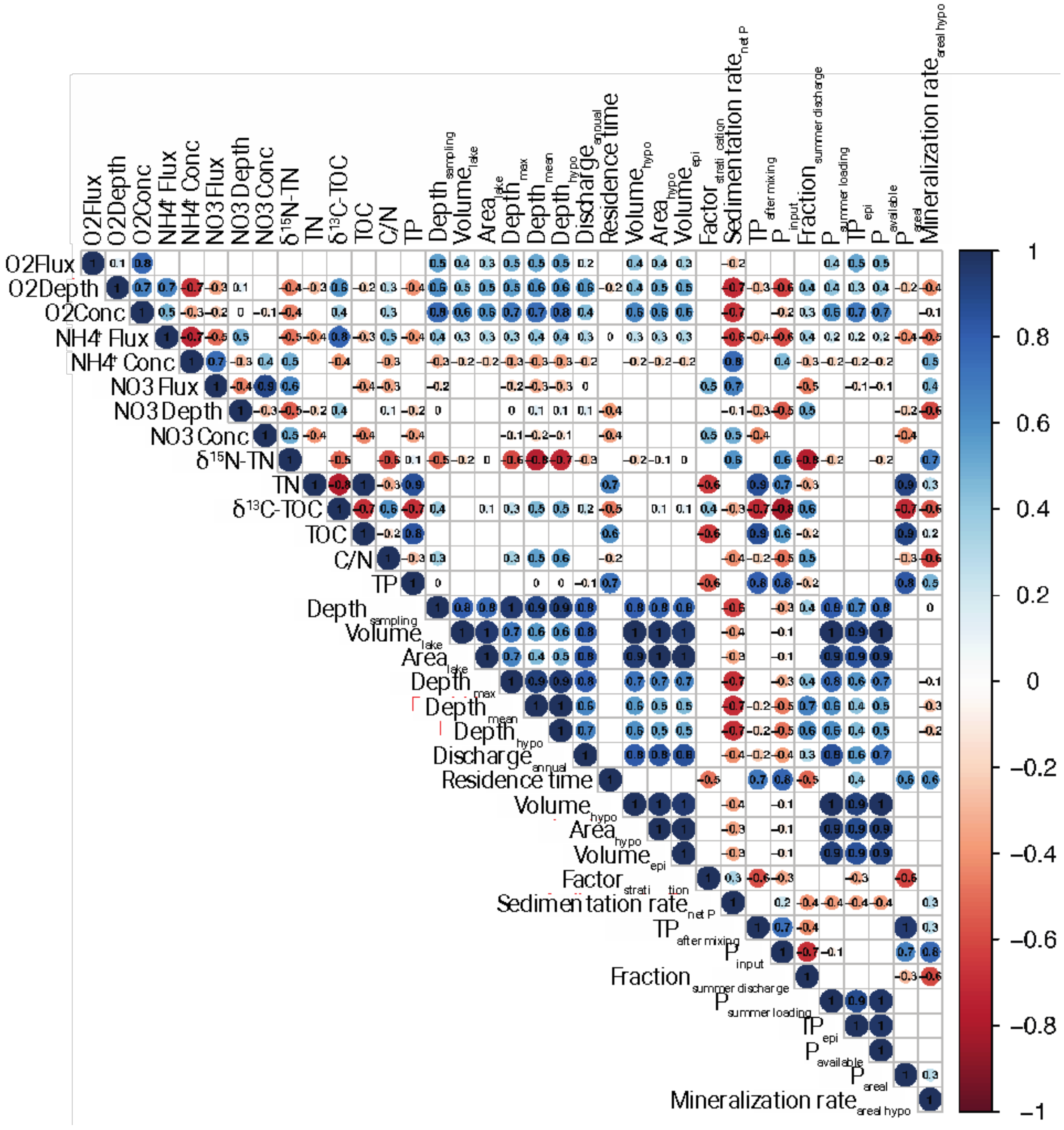

Figure S3: *Correlation plot of the chemical, organic matter (obtained within this project), and lake hydromorphological/limnological data. The reported values represent the significant correlation coefficients (confidence level = 0.95). Abbreviations: total nitrogen (TN), total organic carbon (TOC), carbon/nitrogen ratio (CN), total phosphorus (TP), phosphorous (P), epilimnetic (epi), hypolimnetic (hypo). The employed data include (in order of appearance): fluxes, penetration depths and concentrations of O_2, NH_4^+ and NO_3^-, δ^{15}N-TN, TN content, δ^{13}C-TOC, TOC content, C/N, TP, sampling depth, lake volume, lake area, maximum lake depth, mean lake depth, mean hypolimnion depth (epilimnion = 15 m), annual water discharge, water residence time, volume of hypolimnion, lake area at 15 m depth, epilimnion volume top 15 m, stratification factor (annual average concentration of TP at the surface divided by TP after spring mixing), P net sedimentation rate, TP concentration after spring mixing, fraction of annual water load that is discharged during summer, P loading in summer, TP content in the epilimnion, available P, areal P supply (TP in the top 15 m), areal hypolimnetic mineralization rate. Some of the site-descriptive parameters (e.g. sedimentation rates), when not determined in this study, were adopted from Müller, 2022.*

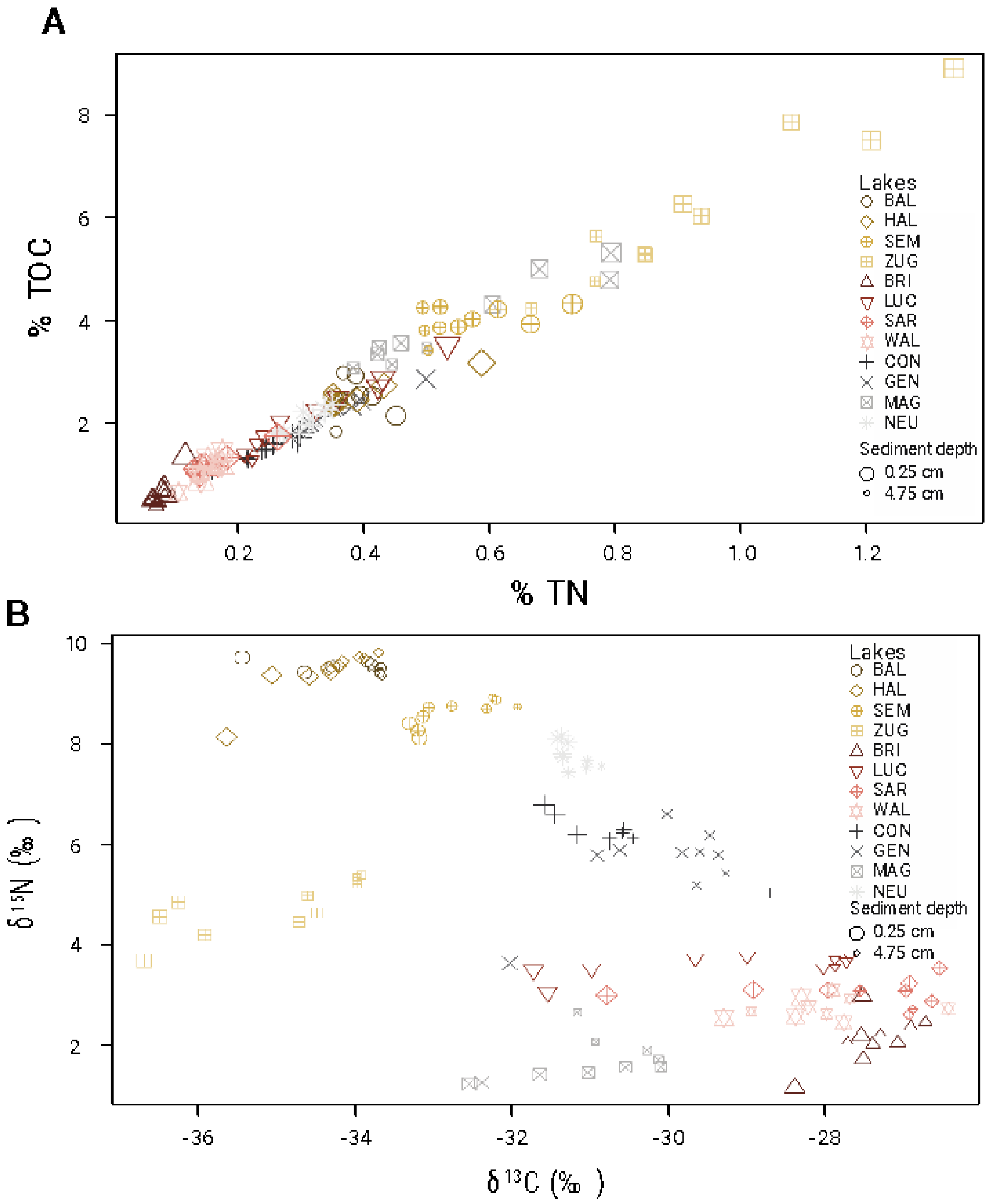

Figure S4: *(A) TOC versus TN contents (in %), (B) bulk organic matter C and N isotopic composition (in ‰) across the top 5 cm of the surface sediments for all twelve lakes: Baldegg (BAL), Brienz (BRI), Constance (CON), Geneva (GEN), Hallwil (HAL), Lucerne (LUC), Maggiore (MAG), Neuchâtel (NEU), Sarnen (SAR), Sempach (SEM), Walen (WAL) and Zug (ZUG). The symbol size decreases with sediment sampling depth from 0.25 cm to 4.75 cm. The color shades are set according to the defined lake groups (yellow shades = agriculture-influenced lakes, red shades = pristine-alpine lakes, gray shades = deep-large lakes).*

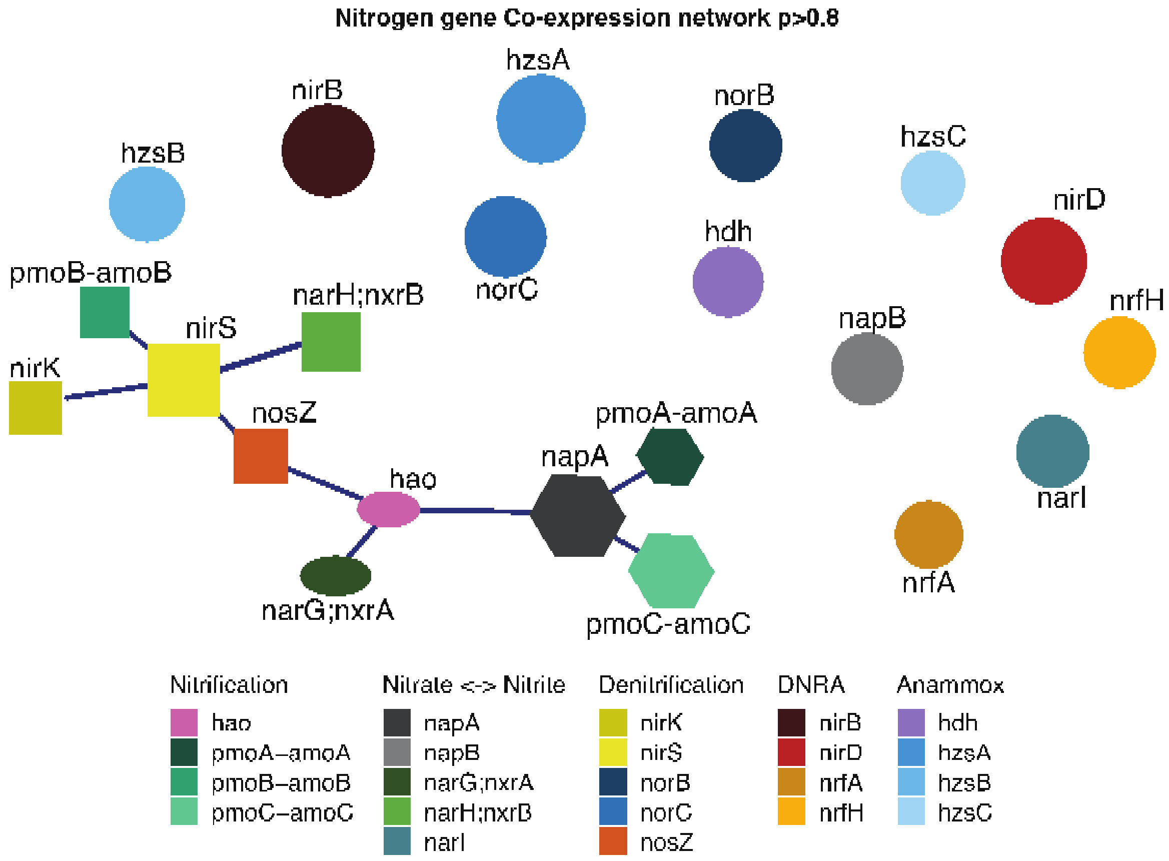

Figure S5: *Network analysis showing the co-expression of operons (color = operon and process, shape = co-expression group). Only edges indicating co-expression with a correlation P>0.8 are shown. Correlations are all positive correlations, indicated by blue lines. Single genes do not have a correlation >0.8 with any other point. The minimum spanning tree was calculated using Prim's algorithm, and the co-expression clusters were identified using the edge.betweeness.community() command from igraph. The network analysis was run with gene expression data and the environmental parameters.*

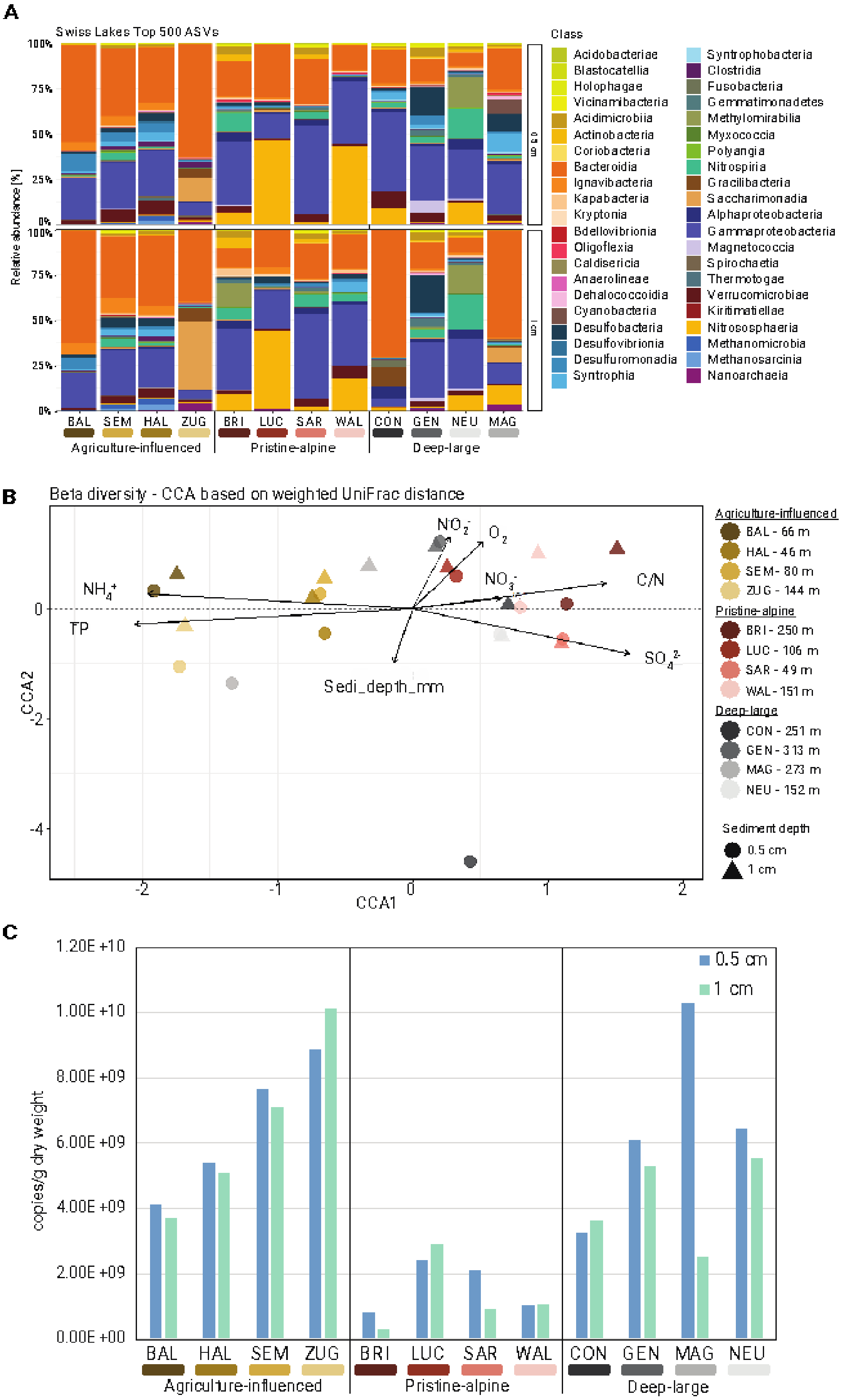

Figure S6: *Microbial community composition on class level (Silva DB138) obtained from 16S rRNA gene amplicon sequencing (A) and analysis of beta diversity (CCA) with the most significantly correlating environmental parameters (p<0.01), explaining the variability (B), and C) the results of the 16S rRNA gene abundance measured by qPCR for each lake and sediment depth. The qPCR triplicates were averaged and normalized to dry sediment mass (g).*

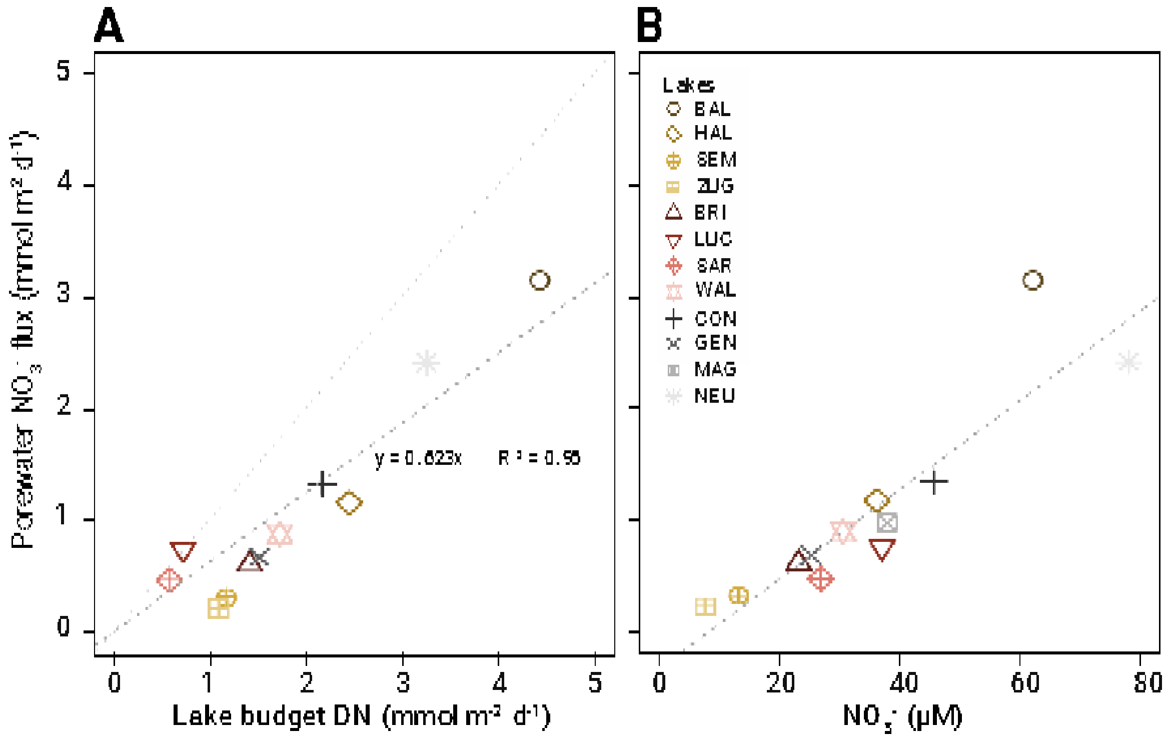

Figure S7: *(A) NO_3^- fluxes and denitrification (DN) rates (in mmol m^{-2} d^{-1}) calculated based on porewater profiles (our study) and whole-lake budgets (Müller, 2022), respectively. The linear regression (dashed grey line) was forced through the origin. The 1:1 line is indicated by the dotted line. The slope of 0.623 in (A) indicates that porewater based fluxes underestimate overall denitrification rates (based on N budget considerations) by approximately 40%; (B) Correlation of NO_3^- fluxes and bottom water concentrations for the twelve lakes included in this study.*

References

[1] Takai K, Horikoshi K. Rapid detection and quantification of members of the archaeal community by quantitative PCR using fluorogenic probes. Appl Environ Microbiol 2000;66:5066–72. https://doi.org/10.1128/AEM.66.11.5066-5072.2000.

[2] Callahan BJ. Dada2 pipeline tutorial n.d. https://benjjneb.github.io/dada2/%0Atutorial.html.

[3] Oksanen J, Kindt R, Legendre P, O'Hara B, Stevens M, Oksanen M, et al. GitHub - vegandevs/vegan: R package for community ecologists: popular ordination methods, ecological null models & diversity analysis n.d. https://github.com/vegandevs/vegan/.

[4] Wickham H. ggplot2. Wiley Interdiscip Rev Comput Stat 2011;3:180–5. https://doi.org/10.1002/wics.147.

[5] McMurdie PJ, Holmes S. Phyloseq: an R Package for reproducible interactive analysis and graphics of microbiome census data. PLoS One 2013;8:61217. https://doi.org/10.1371/journal.pone.0061217.

[6] Ssekagiri A, Sloan W, Ijaz U. microbiomeSeq: An R package for microbial community analysis in an environmental context n.d. https://userweb.eng.gla.ac.uk/umer.ijaz/projects/microbiomeSeq_Tutorial.html.

[7] Paoli L, Ruscheweyh H-J, Forneris CC, Hubrich F, Kautsar S, Bhushan A, et al. Biosynthetic potential of the global ocean microbiome. Nature 2022;607:111–8. https://doi.org/10.1038/s41586-022-04862-3.

[8] Salazar G, Paoli L, Alberti A, Huerta-Cepas J, Ruscheweyh HJ, Cuenca M, et al. Gene expression changes and community turnover differentially shape the global ocean metatranscriptome. Cell 2019;179:1068–83. https://doi.org/10.1016/j.cell.2019.10.014.

[9] Kopylova E, Noé L, Touzet H. SortMeRNA: Fast and accurate filtering of ribosomal RNAs in metatranscriptomic data. Bioinformatics 2012;28:3211–7. https://doi.org/10.1093/bioinformatics/bts611.

[10] Nurk S, Meleshko D, Korobeynikov A, Pevzner PA. MetaSPAdes: a new versatile metagenomic assembler. Genome Res 2017;27:824–34. https://doi.org/10.1101/gr.213959.116.

[11] Hyatt D, Chen G-L, Locascio PF, Land ML, Larimer FW, Hauser LJ. Prodigal: prokaryotic gene recognition and translation initiation site identification. BMC Bioinformatics 2010;11:119. https://doi.org/10.1186/1471-2105-11-119.

[12] Buchfink B, Reuter K, Drost HG. Sensitive protein alignments at tree-of-life scale using DIAMOND. Nat Methods 2021;18:366–8. https://doi.org/10.1038/s41592-021-01101-x.

[13] Li H, Durbin R. Fast and accurate short read alignment with Burrows-Wheeler transform. Bioinformatics 2009;25:1754–60. https://doi.org/10.1093/bioinformatics/btp324.

[14] Ruscheweyh HJ, Milanese A, Paoli L, Karcher N, Clayssen Q, Keller MI, et al. Cultivation-independent genomes greatly expand taxonomic-profiling capabilities of mOTUs across various environments. Microbiome 2022;10:212. https://doi.org/10.1186/s40168-022-01410-z.

[15] Milanese A, Mende DR, Paoli L, Salazar G, Ruscheweyh HJ, Cuenca M, et al. Microbial abundance, activity and population genomic profiling with mOTUs2. Nat Commun 2019;10. https://doi.org/10.1038/s41467-019-08844-4.

[16] Love MI, Huber W, Anders S. Moderated estimation of fold change and dispersion for RNA-seq data with DESeq2. Genome Biol 2014;15. https://doi.org/10.1186/s13059-014-0550-8.
[17] Baumann KBL, Thoma R, Callbeck CM, Niederdorfer R, Schubert CJ, Müller B, et al. Microbial nitrogen transformation potential in sediments of two contrasting lakes is spatially structured but seasonally stable. MSphere 2022;7:e0101321. https://doi.org/10.1128/msphere.01013-21.
[18] Finlay JC, Small GE, Sterner RW. Human influences on nitrogen removal in lakes. Science (1979) 2013;342:247–50. https://doi.org/10.1126/science.1242575.
[19] Müller B, Meyer JS, Gächter R. Denitrification and nitrogen burial in Swiss Lakes. Environ Sci Technol 2022;56:2794–802. https://doi.org/10.1021/acs.est.1c07602.
[20] David MB, Wall LG, Royer T V, Tank JL. Denitrification and the nitrogen budget of a reservoir in an agricultural landscape. Ecological Applications 2006;16:2177–90. https://doi.org/10.1890/1051-0761(2006)016[2177:DATNBO]2.0.CO;2.
[21] Müller B, Thoma R, Baumann KBL, Callbeck CM, Schubert CJ. Nitrogen removal processes in lakes of different trophic states from on-site measurements and historic data. Aquat Sci 2021;83:37. https://doi.org/10.1007/s00027-021-00795-7.
[22] Palacin-Lizarbe C, Camarero L, Hallin S, Jones CM, Catalan J. Denitrification rates in lake sediments of mountains affected by high atmospheric nitrogen deposition. Sci Rep 2020;10:1–9. https://doi.org/10.1038/s41598-020-59759-w.
